# Anti-inflammatory and antioxidant effects of salidroside in diabetic nephropathy: a systematic review and meta-analysis of preclinical studies

**DOI:** 10.3389/fphar.2026.1793037

**Published:** 2026-04-10

**Authors:** Di Sun, Jin Peng, Qi Zhong, Jun Sun, Peng Zhang, Chunwei Wu, Lei Cao, Hailong Chen, Ze He

**Affiliations:** 1 College of Traditional Chinese Medicine, Changchun University of Chinese Medicine, Changchun, China; 2 The Affiliated Hospital of Changchun University of Chinese Medicine, Changchun, China

**Keywords:** animal models, anti-inflammatory, antioxidant, diabetic nephropathy, meta-analysis, salidroside

## Abstract

**Background:**

Salidroside (SAL), a principal bioactive constituent of Rhodiola species, has demonstrated renoprotective potential in diabetic nephropathy (DN). However, the magnitude of SAL’s effects on renal functional outcomes and key mechanistic biomarkers remains unclear.

**Methods:**

This study was conducted in accordance with PRISMA 2020 guidelines. A comprehensive search was performed in PubMed, Embase, Web of Science, the Cochrane Library, and major Chinese databases from inception to 24 November 2025. Preclinical studies evaluating SAL monotherapy in DN animal models were included. Risk of bias was assessed using the SYRCLE tool and summarized in Review Manager (RevMan) 5.4. Meta-analysis was performed using Stata 18.0.

**Results:**

Fourteen studies (257 animals) were included. Pooled estimates suggested SAL was associated with improved renal function and lower blood glucose levels, despite substantial heterogeneity. Specifically, SAL-treated groups exhibited lower serum creatinine (Hedges’ g = −3.83, 95% CI -5.34 to −2.31), blood urea nitrogen (Hedges’ g = −2.90, 95% CI -4.50 to −1.30), and kidney index (Hedges’ g = −2.68, 95% CI -4.71 to −0.65) than controls. SAL also enhanced antioxidant capacity and suppressed inflammatory mediators. For TGF-β1, the pooled estimate did not reach statistical significance and showed heterogeneity, while sensitivity analyses suggested the direction of effect may favor SAL.

**Conclusion:**

SAL provides preliminary preclinical evidence of renoprotection in DN models, potentially by modulating oxidative stress and inflammation. However, interpretation is constrained by high heterogeneity and possible small-study or publication effects. Anti-fibrotic effects, particularly TGF-β1, remain sensitive to methodology, necessitating caution. Rigorous, pre-registered animal trials are required to strengthen the evidence base.

**Systematic Review Registration:**

https://www.crd.york.ac.uk/PROSPERO/view/CRD420251239960, identifier CRD420251239960.

## 1 Introduction

Diabetes mellitus has emerged as a major global public health challenge. Projections from the International Diabetes Federation (IDF) indicate that the population living with diabetes will reach approximately 783 million by 2045 (Sun et al., 2022). As one of the most prevalent microvascular complications of diabetes, diabetic nephropathy (DN) is the leading cause of end-stage renal disease (ESRD) and is associated with a substantial increase in disease burden, impaired quality of life, and escalating healthcare costs worldwide. Clinically, DN is a chronic and progressive disorder characterized by a persistent decline in renal structure and function. Its onset and progression involve multiple interrelated mechanisms, including sustained inflammation, oxidative stress, and renal fibrosis. These processes collectively contribute to progressive functional deterioration. Renal fibrosis is widely regarded as a critical pathological hallmark associated with the progression of DN toward irreversible renal failure (Lin et al., 2018; Wang and Zhang, 2024b).

Currently approved pharmacological strategies for DN management primarily include angiotensin-converting enzyme inhibitors (ACEIs), angiotensin II receptor blockers (ARBs), and sodium-glucose cotransporter 2 (SGLT2) inhibitors (Zhang et al., 2025). Although these agents have demonstrated renoprotective benefits in clinical settings, their long-term use may also be associated with adverse effects, including but not limited to hypotension, urinary tract infections, and ketoacidosis (Lupsa and Inzucchi, 2018; Zhao et al., 2021). Consequently, the identification of novel pharmacological candidates with favorable safety profiles and multi-target renoprotective properties remains an area of active research.

Salidroside (SAL) is a phenethyl glycoside isolated from *Rhodiola* species, a traditional medicinal herb widely used in both Asian and European medicine. Owing to its pleiotropic biological activities and relatively low toxicity, SAL has attracted increasing interest in the prevention and treatment of chronic metabolic diseases. Accumulating preclinical evidence suggests that SAL may exert protective effects in diabetes and its associated complications, including anti-inflammatory and antioxidant actions, as well as anti-fibrotic and anti-apoptotic effects (Zhang et al., 2021). At the molecular level, SAL has been reported to modulate signaling pathways related to mitochondrial function, energy metabolism, and cellular stress responses, which may be involved in DN pathogenesis (Zheng et al., 2019; Chaitanya et al., 2025).

Despite the growing number of preclinical studies investigating the effects of SAL in DN, substantial heterogeneity exists across studies with respect to experimental design, intervention protocols, outcome measures, and reported findings. To date, a systematic and quantitative synthesis of the available preclinical evidence regarding the renoprotective efficacy and mechanistic actions of SAL in DN remains limited. Therefore, the present study performed a systematic review and meta-analysis to quantitatively evaluate the therapeutic effects and underlying mechanisms of SAL in animal models of DN, with a particular focus on renal functional outcomes and key mechanistic pathways related to inflammation, oxidative stress, and fibrosis-related remodeling. This work aims to provide a consolidated overview of the current preclinical evidence base to support future mechanistic investigations and potential translational research. The study protocol was registered in the International Prospective Register of Systematic Reviews (PROSPERO; CRD420251239960).

## 2 Materials and methods

This systematic review and meta-analysis was conducted and reported in accordance with the Preferred Reporting Items for Systematic Reviews and Meta-Analyses (PRISMA, 2020) statement (Page et al., 2021). The study protocol was prospectively registered in the International Prospective Register of Systematic Reviews (PROSPERO; CRD420251239960).

### 2.1 Search strategy

Two reviewers (DS and JP) independently conducted a systematic literature search across eight databases from their inception through 24 November 2025, with no language restrictions. The English databases included PubMed, Embase, Web of Science, and the Cochrane Library, while the Chinese databases included China National Knowledge Infrastructure (CNKI), Wanfang Data, the VIP Database, and the Chinese Biomedical Literature Database (CBM). The search scope encompassed both peer-reviewed journal articles and academic theses.

Our retrieval strategy utilized a combination of controlled vocabulary (such as MeSH and EMTREE) and free-text terms. The search strings were built around keywords for “salidroside”, “diabetic nephropathy”, and animal descriptors including “rat”, “mouse”, or “rodent”. By deliberately omitting specific outcome terms from these strings, we maintained high sensitivity to ensure that eligible studies were not overlooked due to incomplete indexing. Manual screening of the reference lists from all included studies and relevant reviews was also performed. Potential data overlap between theses and journal articles was rigorously cross-checked, and in such cases, only the peer-reviewed article was retained. Detailed search strings are documented in Supplementary Table S1.

### 2.2 Inclusion and exclusion criteria

Eligibility criteria were defined according to the PICO (Participants, Intervention, Comparison, Outcomes) framework.

Participants (P): DN animal models with at least one indicator of renal involvement (albuminuria/urinary protein elevation, Scr/BUN changes, and/or histopathological renal injury) as reported in the original studies, regardless of type 1 or type 2 diabetes induction methods.

Intervention (I): Administration of SAL as monotherapy, regardless of dosage, route of administration, or treatment duration.

Comparison (C): DN animal models receiving vehicle treatment or no pharmacological intervention.

Outcomes (O): The primary outcomes included blood glucose (BG), serum creatinine (Scr), blood urea nitrogen (BUN), and kidney index (KI).

Studies were excluded if they met any of the following criteria:

1. Reviews, editorials, conference abstracts, case reports, clinical studies, or *in vitro* experiments;
2. Full texts were unavailable or relevant data could not be obtained after reasonable attempts;
3. SAL was not used as a single intervention or was administered in combination with other treatments;
4. Duplicate publications or studies with overlapping data;
5. Insufficient quantitative data for meta-analysis, including absence of measures of variability such as standard deviation or standard error.

### 2.3 Data extraction

All retrieved records were imported into EndNote 21 for deduplication, and duplicate records were removed prior to screening. Two reviewers independently screened titles and abstracts to identify potentially eligible studies, after which full-text articles were assessed according to the predefined inclusion and exclusion criteria. Data extraction was independently performed by the same two reviewers using a standardized extraction form; disagreements were resolved by discussion, with adjudication by a third reviewer when consensus could not be reached.

The following information was extracted from each included study:

1. first author and publication year;
2. animal characteristics, including species, sex, sample size, age and body weight;
3. DN model establishment methods and criteria for successful modeling;
4. details of the SAL intervention, including route of administration, dosage, and treatment duration;
5. outcome measures relevant to the meta-analysis.

Data extracted from figures using WebPlotDigitizer (version 4.5) were independently digitized by two reviewers. Each graph was digitized twice, and discrepancies were resolved by consensus; when discrepancies were within a prespecified tolerance, the averaged value was used. Data reported in unclear formats were verified against the full text and supplementary materials, and corresponding authors were contacted when necessary.

To avoid unit-of-analysis errors, we included only one SAL–control comparison per study for each outcome (one SAL arm vs. one model-control arm). When multiple SAL arms were available, we pre-specified selecting one arm (the intermediate dose when three doses were reported; the higher dose when two doses were reported). When multiple time points were reported, the final time point was used. As a sensitivity analysis, we alternatively extracted the highest dose in studies that reported three dose levels.

If outcome data were reported as the standard error of the mean (SEM), they were converted to standard deviation (SD) using the following formula: SD = SEM × $\sqrt{N}$ , where n is the sample size.

### 2.4 Risk-of-bias assessment

The methodological quality of the included studies was assessed using the SYRCLE risk-of-bias tool for animal studies (Hooijmans et al., 2014). Two reviewers independently evaluated each study across the predefined domains, including selection bias, performance bias, detection bias, attrition bias, reporting bias, and other potential sources of bias, with each domain rated as low, unclear, or high risk of bias. Risk-of-bias assessments were performed using Review Manager (RevMan) software version 5.4. Disagreements were resolved through discussion; if consensus could not be reached, a third reviewer was consulted to adjudicate.

### 2.5 Statistical analysis

All analyses were conducted in Stata 18.0. Continuous outcomes were pooled as standardized mean differences (Hedges’ g) using random-effects models, with between-study variance estimated by restricted maximum likelihood (REML). Confidence intervals were calculated using the Hartung–Knapp–Sidik–Jonkman (HKSJ) method with a t-distribution. Heterogeneity was tested using Cochran’s Q (P < 0.10) and quantified using I^2^, and prediction intervals were reported for primary outcomes to reflect the range of treatment effects that might be expected in a future study when between-study heterogeneity is substantial.

Robustness was evaluated using leave-one-out analyses. Baujat-type influence diagnostics were applied to the primary outcomes and to outcomes showing very large effects (SMD ≥4). When influential studies were identified, influential-study-excluded analyses were performed. In addition, sensitivity analyses excluding studies with very small group sizes (n ≤ 8 per arm) were conducted for the primary outcomes and for outcomes with SMD ≥4. For the primary outcomes and for outcomes with SMD ≥4, pooled effects were presented as SMDs and, when units were comparable after harmonization, as pooled raw mean differences (MDs) reported side by side. For outcomes with SMD ≥4, study-level raw MDs were additionally tabulated to contextualize unusually large standardized effects.

Prespecified subgroup analyses explored heterogeneity by species or strain, diabetic nephropathy model type, dose category, treatment duration, and reported purity of SAL. Subgroup analyses were conducted only when at least three comparisons were available within a subgroup. All included studies administered SAL orally; therefore, subgroup analysis by administration route was not feasible. SAL source and purity were extracted when reported and summarized in the Supplementary Table S2. Urinary protein outcomes were stratified by reporting metric (24-h collection/excretion-based vs. concentration-based). Mechanistic biomarkers were stratified by sample source and, when feasible, by assay method. For outcomes with twelve or more comparisons, univariable random-effects meta-regression was conducted using consistently reported covariates. Ecological meta-regression relating glucose change to renal outcomes was performed to examine potential confounding by glycemic control, supplemented by exploratory subgroup analysis based on median glucose reduction. Planned analyses stratified by treatment timing and diabetes-induction severity were limited by sparse data; therefore, sensitivity analyses excluding the single preventive-design study and studies using lower induction thresholds were conducted. Publication bias was evaluated using funnel plots and, when sufficient data were available (≥10), Egger’s and Begg’s tests, with trim-and-fill applied as a sensitivity analysis when asymmetry was suspected.

## 3 Results

### 3.1 Study selection

**FIGURE 1:**
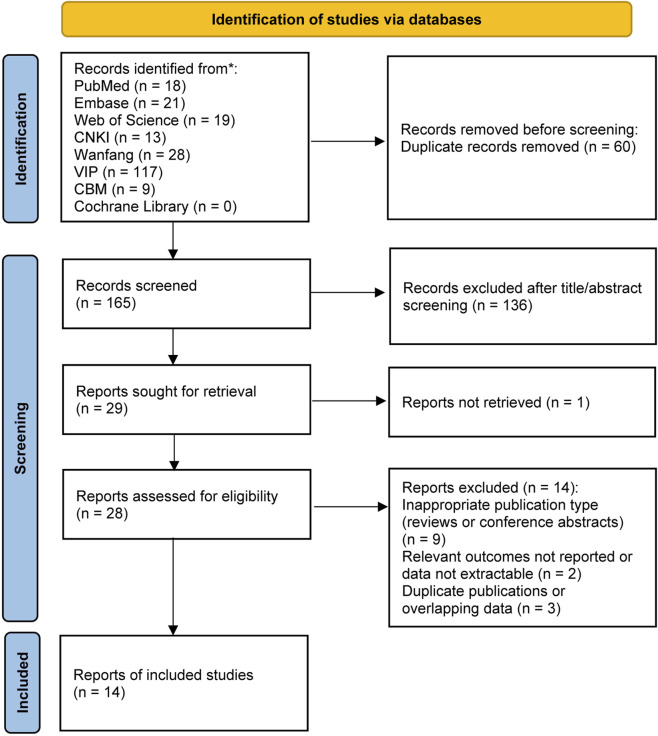
Flow diagram of the study selection process.

The study selection process is illustrated in Figure 1. A total of 225 records were identified through electronic database searches, including PubMed (n = 18), Embase (n = 21), Web of Science (n = 19), Cochrane Library (n = 0), CNKI (n = 13), CBM (n = 9), Wanfang Data (n = 28), and VIP (n = 117). After removal of duplicates, 165 records remained and were screened based on titles and abstracts, of which 136 records were excluded as irrelevant. Subsequently, 29 articles were retrieved for full-text assessment. Among them, one article could not be obtained in full text and was therefore excluded. The full texts of the remaining 28 articles were assessed for eligibility. Of these, fourteen articles were excluded after full-text review because they did not meet the predefined inclusion criteria. Ultimately, 14 studies met the predefined inclusion criteria and were included in the final meta-analysis.

### 3.2 Characteristics of included studies

A total of 14 studies involving 257 diabetic animals were included in the meta-analysis, with 129 animals allocated to the SAL-treated groups and 128 to the DN model control groups. Regarding species and strains, Wistar rats were utilized in four studies (75/257, 29.2%), Sprague-Dawley rats in six studies (110/257, 42.8%), C57BLKS/J mice in one study (18/257, 7.0%), and C57BL/6J mice in three studies (54/257, 21.0%); all included studies were conducted exclusively on male animals. Data regarding animal body weight and age were reported in 10 and 9 studies, respectively.

Regarding the induction of the DN model, streptozotocin (STZ) was administered alone in seven studies, STZ combined with unilateral nephrectomy was used in two studies, and STZ combined with a high-fat or high-sugar/high-fat diet was employed in four studies. One study utilized a spontaneous diabetes model. The duration of the SAL intervention ranged from 4 to 12 weeks, with dosages varying from 20 to 200 mg/kg/day. Primary outcomes included blood glucose (BG), serum creatinine (Scr), blood urea nitrogen (BUN), and kidney index (KI), while secondary outcomes comprised 24-h urinary protein (24-h UP) excretion and body weight. Additionally, several studies reported lipid metabolism parameters (total cholesterol and triglycerides) and mechanistic markers related to inflammation (IL-1β, TNF-α), oxidative stress (SOD, MDA), and fibrosis (TGF-β1). Detailed characteristics of the included studies are summarized in Table 1.

**TABLE 1:** Baseline characteristics of the included studies.

Study (year)	Species	Sex	Sample size (SAL/control)	DN induction method and criteria for successful DN induction	SAL intervention (route, dose, duration)	Outcomes
An et al. (2025)	C57BL/6 J mice	Male	10/10	STZ (i.p. 150 mg/kg) BG ≥ 11.1 mmol/L	Oral gavage 50/100/150 mg/kg 12 weeks	Scr, BUN, KI, 24-h UP, BG, TNF-α
Guo et al. (2018)	Wistar rat	Male	8/7	STZ (i.p. 45 mg/kg), right nephrectomy BG ≥ 16.7 mmol/L	Oral gavage 70 mg/kg 8 weeks	Scr, BUN, KI, 24-h UP, BW, TIS
Leng et al. (2019)	SD rat	Male	12/12	STZ (i.p. 40 mg/kg), right nephrectomy BG ≥ 16.7 mmol/L	Oral gavage 50/100/200 mg/kg 12 weeks	BUN, KI, 24-h UP, SOD, MDA, TIS
Leng et al. (2024)	C57BL/6 J mice	Male	12/12	HFD + STZ (i.p. 50 mg/kg) FBG >11.1 mmol/L, positive urine microalbumin	Oral gavage 20/100 mg/kg 10 weeks	Scr, BUN, KI, 24-h UP, TC, TG, SOD, MDA
Li et al. (2024)	SD rat	Male	8/8	HFD + STZ (i.p. 35 mg/kg) FBG ≥16.7 mmol/L, 24-h urinary protein ≥30 mg	Oral gavage 80 mg/kg 4 weeks	Scr, BUN, TC, TG, IL-1β
Pei et al. (2022)	Wistar rat	Male	8/8	STZ (i.p. 65 mg/kg) BG ≥ 16.7 mmol/L, urine protein >30 mmol/L	Oral gavage 50/100 mg/kg 8 weeks	Scr, BUN, 24-h UP, BW, TC, TG, IL-1β, TNF-α, SOD, MDA, MFA
Piao et al. (2017)	SD rat	Male	8/8	STZ (i.p. 60 mg/kg) BG > 16.7 mmol/L, urine protein >30 mg/kg/day	Oral gavage 100 mg/kg 10 weeks	BG, Scr, BUN, IL-1β, TNF-α, SOD, MDA
Qi et al. (2021)	SD rat	Male	12/12	STZ (i.p. 45 mg/kg) BG > 16.7 mmol/L, positive urine protein	Oral gavage 10/20 mg/kg 8 weeks	Scr, BUN, KI, BG, BW, IL-1β, TNF-α, SOD, MDA, MFA
Qin et al. (2025)	SD rat	Male	10/10	HFD + STZ (i.p. 30 mg/kg) BG > 16.7 mmol/L, urine protein >30 mg/kg/day	Oral gavage 20 mg/kg 16 weeks	Scr, BUN, BG, TC, IL-1β
(Shati and Alfaifi, 2020) (A)	Wistar rat	Male	12/12	STZ (i.v. 65 mg/kg) BG > 16.7 mmol/L	Oral gavage 100 mg/kg 8 weeks	Scr, BUN, KI, 24-h UP, BG, BW, MDA
(Shati, 2020) (B)	Wistar rat	Male	10/10	STZ (i.p. 65 mg/kg) BG > 16.7 mmol/L	Oral gavage 100 mg/kg 8 weeks	Scr, 24-h UP, BG, BW
Wu et al. (2016)	C57BLKS/J mice	Male	9/9	Spontaneous diabetes BG ≥ 16.7 mmol/L	Oral gavage 25/50/100 mg/kg 11 weeks	Scr, BG, BW
Xue et al. (2019)	C57BL/6 J mice	Male	5/5	HFD + STZ (i.p. 40 mg/kg) BG > 16.7 mmol/L	Oral gavage 50/100 mg/kg 10 weeks	Scr, BUN, KI, BG, TC, TG
Zhang (2014)	SD rat	Male	5/5	STZ (i.p. 65 mg/kg) BG ≥ 16.7 mmol/L, urine glucose ≥3+	Oral gavage 30 mg/kg 12 weeks	Scr, BUN, KI, 24-h UP, BW

Abbreviations: 24-h UP, 24-h urinary protein; BUN, blood urea nitrogen; BW, body weight; DN, diabetic nephropathy; FBG, fasting blood glucose; HFD, high-fat diet; IL-1β, interleukin-1β; i. p., intraperitoneal; KI, kidney index; MDA, malondialdehyde; MFA, Masson’s trichrome–quantified fibrotic area (%); SAL, salidroside; Scr, serum creatinine; SOD, superoxide dismutase; STZ, streptozotocin; TC, total cholesterol; TIS, semi-quantitative tubular injury score; TGF-β1, transforming growth factor-β1; TG, triglycerides; TNF-α, tumor necrosis factor-α.

(A) and (B) indicate two different studies published in 2020 by the same first author and are used to distinguish them in the table.

### 3.3 Risk of bias

**FIGURE 2:**
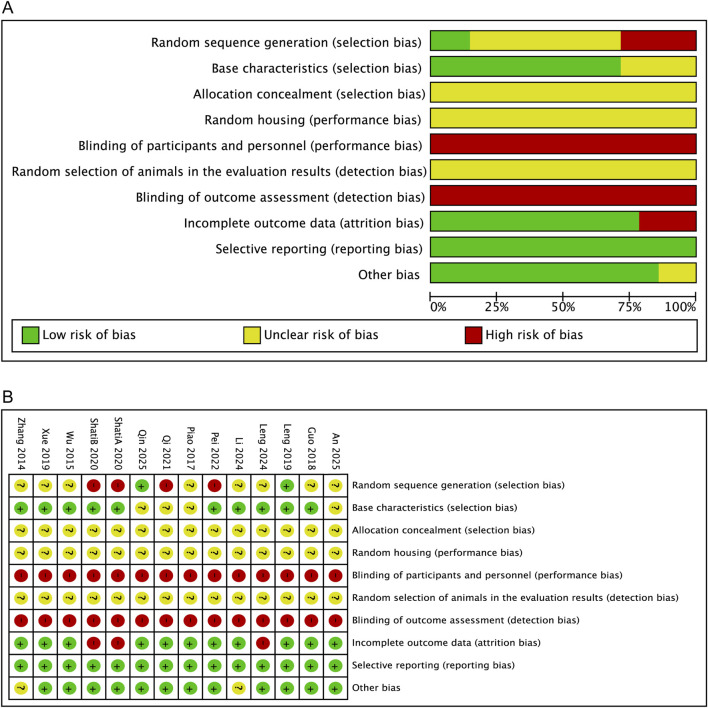
Risk-of-bias assessment of the included studies using the SYRCLE tool **(A)** Risk-of-bias graph **(B)** Risk-of-bias summary.

The risk of bias assessment for the included studies is presented in Figure 2. Regarding selection bias, eight studies reported randomization without specifying the methods, two studies clearly described random allocation, and four studies were judged to be at high risk of bias for random sequence generation; baseline characteristics were comparable in ten studies, while four studies did not provide relevant information. None of the fourteen studies reported allocation concealment or random housing. For detection bias, none of the studies reported random selection of animals for outcome assessment, and outcome assessment was not blinded in any study. Eleven studies were judged to be at low risk of attrition bias, while three studies showed a high risk due to incomplete outcome data. No additional sources of bias were identified based on the information available from the included studies.

### 3.4 Meta-analysis

#### 3.4.1 Primary outcomes

*P* values denote heterogeneity (Q-test) unless otherwise stated.

##### 3.4.1.1 Effect of SAL on BG

**FIGURE 3:**
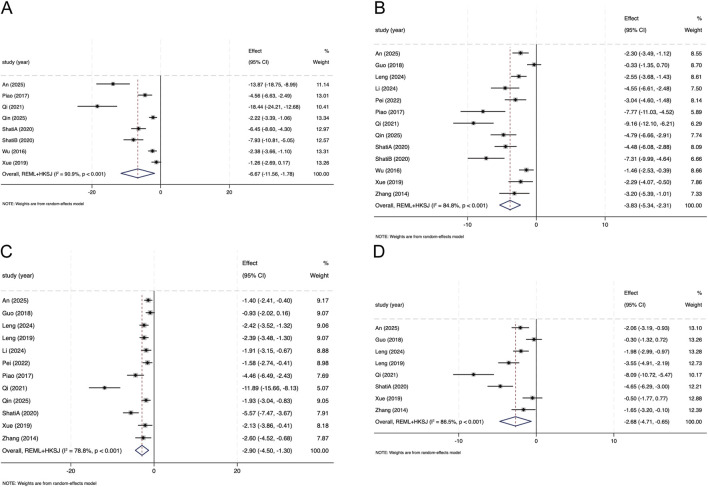
Forest plots illustrating the effects of salidroside on **(A)** blood glucose (BG) **(B)** serum creatinine (Scr) **(C)** blood urea nitrogen (BUN), and **(D)** kidney index (KI) in animal models of diabetic nephropathy.

Eight studies reported BG. Meta-analysis suggested lower BG in the SAL group than in DN model controls (Hedges’ g = −6.67, 95% CI -11.56 to −1.78; I^2^ = 90.9%, *P* < 0.001; Figure 3A). Influence diagnostics (leave-one-out and Baujat-type) identified (Qi et al., 2021) and (An et al., 2025) as influential studies; omitting these studies reduced the pooled estimate, while the direction remained consistent (Hedges’ g = −3.93, 95% CI −6.63 to −1.22; I^2^ = 84.2%; Supplementary Figures S1A, S2A; Supplementary Table S3). The 95% prediction interval (PI) was −20.82 to 7.47.

##### 3.4.1.2 Effect of SAL on Scr.

Thirteen studies reported Scr. Meta-analysis suggested lower Scr in the SAL group than in DN model controls (Hedges’ g = −3.83, 95% CI -5.34 to −2.31; I^*2*^ = 84.8%, *P* < 0.001; Figure 3B). Influence diagnostics identified (Qi et al., 2021) as influential; omitting it attenuated the pooled estimate without changing its direction (Supplementary Figures S1B, S2B; Supplementary Table S3). The 95% PI was −8.95 to 1.29.

##### 3.4.1.3 Effect of SAL on BUN

Twelve studies reported BUN. Meta-analysis suggested lower BUN in the SAL group than in DN model controls (Hedges’ g = −2.90, 95% CI -4.50 to −1.30; I^2^ = 78.8%, P < 0.001; Figure 3C). Influence diagnostics identified (Qi et al., 2021) as influential; omitting it attenuated the pooled effect and reduced heterogeneity (Hedges’ g = −2.29, 95% CI −3.12 to −1.46; I^2^ = 61.7%; Supplementary Figures S1C, S2C; Supplementary Table S3), without changing the direction of effect. The 95% PI was −7.63 to 1.83.

##### 3.4.1.4 Effect of SAL on KI

Eight studies reported KI. Meta-analysis suggested lower KI in the SAL group than in DN model controls (Hedges’ g = −2.68, 95% CI -4.71 to −0.65; I^2^ = 86.5%, *P* < 0.001; Figure 3D). Influence diagnostics identified (Qi et al., 2021) as influential; omitting it attenuated the pooled estimate while maintaining the same direction (Supplementary Figures S1D, S2D; Supplementary Table S3). The 95% PI was −8.42 to 3.05.

#### 3.4.2 Secondary outcomes

##### 3.4.2.1 Effect of SAL on 24-h UP

**FIGURE 4:**
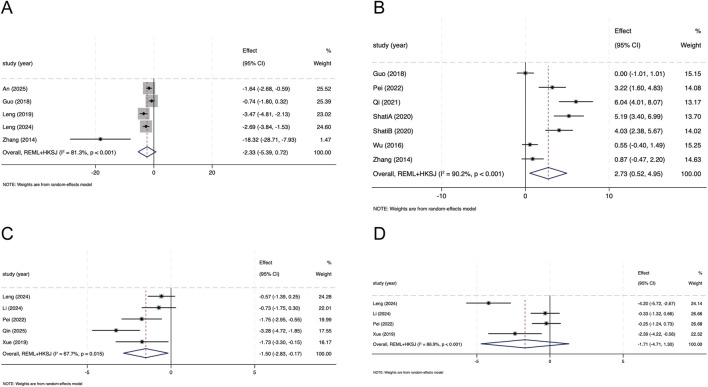
Forest plots illustrating the effects of salidroside on **(A)** 24-h urinary protein (24-h UP) **(B)** body weight (BW) **(C)** total cholesterol (TC), and **(D)** triglycerides (TG) in animal models of diabetic nephropathy.

Eight studies reported 24-h UP excretion. Meta-analysis suggested a trend toward lower 24-h UP with SAL than in DN model controls, although the effect did not reach statistical significance (Hedges’ g = −2.33, 95% CI −5.39 to 0.72; I^2^ = 81.3%, *P* < 0.001; Figure 4A). As a sensitivity analysis addressing the inclusion of non–24-h measures, pooling all UP outcomes (including both 24-h excretion and concentration-based measures) suggested lower UP with SAL (Hedges’ g = −2.78, 95% CI −4.43 to −1.13; I^2^ = 77.3%; Supplementary Figure S3A). Stratified analyses by reporting metric showed markedly lower heterogeneity among concentration-based measures (Hedges’ g = −3.40, 95% CI −5.66 to −1.14; I^2^ = 27.1%; Supplementary Figure S3B). The direction of effect was consistent across measurement types.

##### 3.4.2.2 Effect of SAL on body weight

Seven studies reported body weight outcomes. Meta-analysis suggested higher body weight with SAL than in DN model group (Hedges’ g = 2.73, 95% CI 0.52 to 4.95; I^2^ = 90.2%, *P* < 0.001; Figure 4B). Between-study heterogeneity was substantial.

##### 3.4.2.3 Effect of SAL on lipid parameters

Five studies reported total cholesterol (TC). Meta-analysis suggested lower TC with SAL than in the DN model group (Hedges’ g = −1.50, 95% CI -2.83 to −0.17; I^2^ = 67.7%, *P* = 0.008; Figure 4C). Four studies reported triglycerides (TG). Meta-analysis did not show clear differences in TG between groups (Hedges’ g = −1.71, 95% CI -4.71 to 1.30; I^2^ = 86.9%, *P* < 0.001; Figure 4D). Between-study heterogeneity was substantial.

#### 3.4.3 Association between glucose lowering and renal outcomes

To assess potential confounding by glycemic control, we conducted study-level random-effects meta-regression relating glucose change to renal outcomes. No clear linear association was observed for Scr (k = 8; P = 0.22) or BUN (k = 6; P = 0.14). Median-split subgroup analyses by glucose reduction yielded effects in the same direction in both strata, with no between-subgroup differences for Scr (Q_b P = 0.37) or BUN (Q_b P = 0.27) (Supplementary Figure S4; Supplementary Table S4). Only two comparisons reported both urinary protein and blood glucose (k = 2), precluding quantitative assessment; these were summarized descriptively (Supplementary Table S4). Sensitivity analyses excluding the preventive-design study and studies using a lower diabetes-induction threshold did not materially change the direction of pooled estimates for key outcomes (Supplementary Table S5).

#### 3.4.4 Mechanistic outcomes

Influence (leave-one-out) analyses identified a small number of potentially influential studies for several mechanistic outcomes; outlier-omitted sensitivity analyses yielded consistent directions of effect (Supplementary Figures S5, S6; Supplementary Table S6). To reduce conceptual heterogeneity, mechanistic biomarkers were stratified by sample source (renal tissue/cortex vs. serum) and, when feasible, by assay type (WB vs. IHC), with stratified results summarized in Supplementary Table S7. Strata containing only one study (k = 1) were reported descriptively (not pooled) and should be considered exploratory.

##### 3.4.4.1 Effect of SAL on inflammatory biomarkers

**FIGURE 5:**
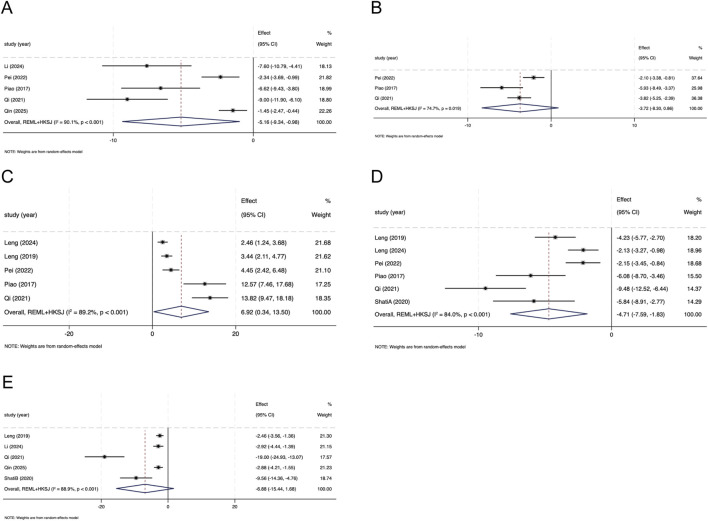
Forest plots illustrating the effects of salidroside on **(A)** interleukin-1β (IL-1β) **(B)** tumor necrosis factor-α (TNF-α) **(C)** superoxide dismutase (SOD) **(D)** malondialdehyde (MDA), and **(E)** transforming growth factor-β1 (TGF-β1) in animal models of diabetic nephropathy.

Five studies reported IL-1β. Meta-analysis suggested lower IL-1β with SAL than in the DN model group (Hedges’ g = −5.16, 95% CI -9.34 to −0.98; I^2^ = 90.1%, *P* < 0.001; Figure 5A). Influence diagnostics (leave-one-out and Baujat-type) identified (Qi et al., 2021) as influential; excluding it attenuated the pooled estimate (Hedges’ g = −4.21, 95% CI −9.04 to 0.61; I^2^ = 86.5%; Supplementary Figures S5, S6; Supplementary Table S6). For serum IL-1β, the pooled estimate remained in the same direction (Hedges’ g = −6.18, 95% CI −10.92 to −1.43; I^2^ = 87.9%; Supplementary Table S7).

Three studies reported TNF-α. Meta-analysis did not show clear differences in TNF-α between groups (Hedges’ g = −3.72, 95% CI -8.30 to 0.86; I^2^ = 74.7%, *P* = 0.019; Figure 5B). Stratified analyses for TNF-α were not performed because further stratification would yield sparse strata (k = 3).

Two studies reported IL-18. Given the small number of studies (k = 2), quantitative pooling was not performed; both studies reported lower IL-18 levels with SAL than in DN model controls.

##### 3.4.4.2 Effect of SAL on oxidative stress-related markers

Five studies reported SOD. Meta-analysis suggested higher SOD with SAL than in the DN model group (Hedges’ g = 6.92, 95% CI 0.34 to 13.50; I^2^ = 89.2%, *P* < 0.001; Figure 5C). Influence diagnostics identified (Qi et al., 2021) and (Piao et al., 2017) as influential; excluding these studies reduced the pooled estimate while maintaining the direction (Hedges’ g = 3.23, 95% CI 0.93 to 5.54; I^2^ = 33.3%; Supplementary Figures S5, S6; Supplementary Table S6). Among renal tissue/cortex measures, the pooled estimate was imprecise (Hedges’ g = 7.95, 95% CI −1.13 to 17.03; I^2^ = 91.8%; Supplementary Table S7).

Six studies reported MDA. Meta-analysis suggested lower MDA with SAL than in DN model controls (Hedges’ g = −4.71, 95% CI −7.59 to −1.83; I^2^ = 84.0%, P < 0.001; Figure 5D). Influence diagnostics identified (Qi et al., 2021) as influential; excluding it attenuated the pooled estimate while maintaining the direction (Hedges’ g = −3.75, 95% CI −6.06 to −1.44; I^2^ = 73.2%; Supplementary Figures S5, S6; Supplementary Table S6). Among renal tissue/cortex measures, the pooled estimate remained in the same direction (Hedges’ g = −4.89, 95% CI −8.72 to −1.06; I^2^ = 86.6%; Supplementary Table S7).

##### 3.4.4.3 Effect of SAL on fibrotic and histopathological outcomes

Five studies reported transforming growth factor-β1 (TGF-β1). Meta-analysis yielded an imprecise pooled estimate with substantial heterogeneity (Hedges’ g = −6.88, 95% CI -15.44 to 1.68; I^2^ = 88.9%, *P* < 0.001; Figure 5E). Influence diagnostics identified (Qi et al., 2021) as influential; excluding it attenuated the pooled estimate and reduced heterogeneity (Hedges’ g = −2.86, 95% CI −4.80 to −0.92; I^2^ = 62.5%; Supplementary Figures S5, S6; Supplementary Table S6). In the WB subgroup (k = 4), the pooled estimate remained in the same direction (Hedges’ g = −2.86, 95% CI −4.80 to −0.92; Supplementary Table S7), and after removing one outlier within the WB subgroup, the estimate was similar with no residual heterogeneity (Hedges’ g = −2.70, 95% CI −3.36 to −2.04; I^2^ = 0%; Supplementary Table S7).

Two studies evaluated fibrosis area quantified by Masson staining. Given the small number of studies (k = 2), quantitative pooling was not performed; both studies reported smaller fibrosis area with SAL than in DN model controls.

Two studies evaluated tubular injury using semi-quantitative scoring. Given the small number of studies (k = 2), quantitative pooling was not performed; both studies reported lower tubular injury scores with SAL than in DN model controls.

##### 3.4.4.4 Summary of mechanistic findings

Collectively, meta-analyses suggested lower IL-1β, TNF-α, MDA, and TGF-β1, and higher SOD, in SAL-treated animals compared with the DN model group. Given the substantial heterogeneity and the limited number of studies for several biomarkers, these findings should be interpreted as supporting mechanistic hypotheses rather than establishing causality. Stratification by sample source and assay type generally yielded consistent directions where data were available; however, several strata were informed by single studies, limiting quantitative inference and warranting cautious interpretation.

### 3.5 Sensitivity analysis and influence diagnostics

To evaluate robustness in outcomes with extreme SMDs, we conducted influence diagnostics using Baujat-type and leave-one-out analyses. Omitting influential comparisons generally attenuated pooled magnitudes while preserving effect directions (Supplementary Figures S1, S2, S5, S6; Supplementary Tables S2, S6). For the primary outcomes, pooled effects decreased from g = −6.67 to −3.93 (BG), −3.83 to −3.40 (Scr), −2.90 to −2.29 (BUN), and −2.68 to −2.04 (KI) (Supplementary Table S3). Similar attenuation was observed for selected biomarkers (e.g., SOD: 6.92 to 3.23; MDA: −4.71 to −3.75), with heterogeneity reduced for some outcomes (e.g., SOD I^2^: 89.2%–33.3%) (Supplementary Table S6). For IL-1β, the estimate remained directionally consistent but became imprecise after removing the influential study (from g = −5.16 [−9.34, −0.98] to −4.21 [−9.04, 0.61]) (Supplementary Table S6).

To aid interpretation of extreme SMDs, we additionally reported study-level raw MDs in original units alongside Hedges’ g where available (Table 2; Supplementary Table S8). For outcomes with harmonized units, we also pooled MDs, which were generally directionally consistent with SMD-based analyses (Supplementary Table S9). Excluding very small group sizes (n ≤ 8) and substituting the highest-dose arm in multi-arm studies did not change the direction of pooled estimates (Supplementary Tables S10, S11). These observations suggest that although the magnitude of pooled effects may be influenced by small-study characteristics, the direction of treatment effects appears broadly consistent across experimental models.

**TABLE 2:** Selected study-level raw mean differences (MDs) in original units for outcomes with unusually large standardized effects.

Outcome	Study (year)	SAL mean ± SD (n)	Control mean ± SD (n)	Raw MD	SMD (Hedges’ g)	Key driver
BG	An et al. (2025)	21.9 ± 0.44 (10)	28.7 ± 0.50 (10)	−6.82	−13.88	Very small SD
IL-1β	Qin et al. (2025)	24.37 ± 4.28 (10)	31 ± 4.45 (10)	−6.63	−5.16	Moderate SD; influential in LOO
SOD	Piao et al. (2017)	32.87 ± 0.46 (8)	27.2 ± 0.39 (8)	5.67	12.57	Very small SD + small n
MDA	Piao et al. (2017)	11.72 ± 0.29 (8)	13.65 ± 0.31 (8)	−1.93	−6.08	Very small SD + small n
TGF-β1	Shati (2020) (B)	0.30 ± 0.05 (6)	0.93 ± 0.07 (6)	−0.63	−8.09	Very small SD + very small n

This excerpt highlights studies with extreme standardized effects; full data are provided in Supplementary Table S8. Very small within-group SDs, and/or very small sample sizes can mechanically inflate Hedges’ g even when raw MDs, are plausible. Complete study-level raw MDs, for all primary and high-SMD, outcomes are provided in Supplementary Table S8.

### 3.6 Subgroup analysis

Subgroup analyses by dose (<100 vs. ≥100 mg/kg) and treatment duration (<10 vs. ≥10 weeks) showed no clear between-subgroup differences for BG, Scr, BUN, or KI (all P for subgroup differences >0.05; Supplementary Table S12.1, 12.2). For BUN, heterogeneity was lower in the ≥10-week subgroup than in the <10-week subgroup (Supplementary Table S12.2). Species stratification indicated a difference for Scr between rats and mice (P = 0.005), with lower heterogeneity among mouse studies than rat studies; BG, BUN, and KI showed no clear between-species differences (Supplementary Table S12.3). Subgroup analyses by DN modeling method and animal strain were limited by sparse data in several categories and were pooled only for strata with ≥3 studies per outcome (Supplementary Table S12.4, 12.5). Purity reporting (reported ≥95% vs. not reported) was not associated with subgroup differences for any primary outcome (all P for subgroup differences >0.05; Supplementary Table S12.6).

For Scr (k = 13) and BUN (k = 12), univariable random-effects meta-regression (REML) examined associations of species, DN model type, dose category, and treatment duration with effect size (Supplementary Figure S7; Supplementary Table S13). For Scr, DN model type showed a potential association with effect size (model type 2 vs. 1: β = 1.47; P = 0.032), whereas dose category, species, and duration were not associated (all P > 0.05; Supplementary Table S13). No covariate showed evidence of association with BUN (all P > 0.05), and substantial residual heterogeneity remained (Supplementary Table S13). These findings should be interpreted cautiously given the limited statistical power for some covariate levels and the presence of substantial residual heterogeneity.

### 3.7 Publication bias

**FIGURE 6:**
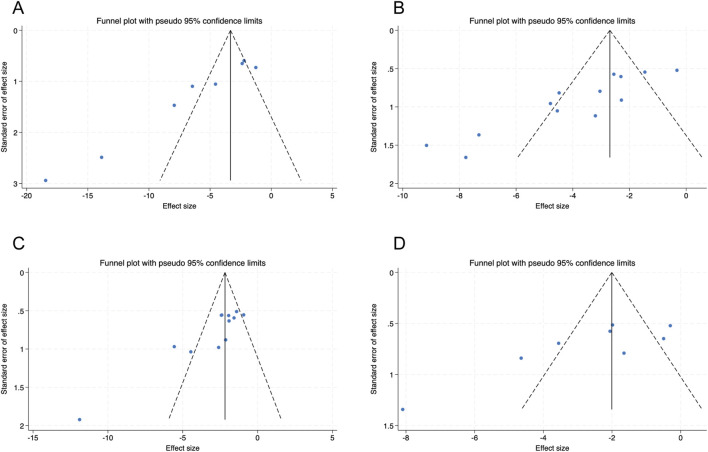
Funnel plots with pseudo–95% confidence limits for **(A)** blood glucose (BG) **(B)** serum creatinine (Scr) **(C)** blood urea nitrogen (BUN), and **(D)** kidney index (KI).

Publication bias was assessed primarily by visual inspection of funnel plots (Figure 6) and, when k ≥ 10, by Begg’s rank correlation test and Egger’s regression test. For Scr (k = 13) and BUN (k = 12), Begg’s and Egger’s tests suggested funnel-plot asymmetry (Supplementary Table S14), which may reflect small-study effects. Trim-and-fill analyses under a random-effects model (REML) imputed no missing studies for either outcome (n = 0), and the adjusted pooled estimates were similar to the main results (Supplementary Table S14). For BG and KI (k = 8 each), formal tests were not performed because of limited power; assessment therefore relied on funnel-plot inspection (Figure 6; Supplementary Table S14). Overall, interpretation is limited by substantial heterogeneity and the small number of studies for several outcomes.

## 4 Discussion

DN remains the leading cause of ESRD worldwide, clinically characterized by persistent albuminuria and a progressive decline in glomerular filtration rate (de Boer et al., 2022). Its progression is characterized by a cascade involving glomerular hypertrophy, mesangial matrix expansion, and tubulointerstitial fibrosis, which ultimately results in glomerulosclerosis and irreversible renal injury (Oshima et al., 2021). Although the precise etiology is multifactorial, current theoretical frameworks increasingly recognize the interplay between oxidative stress, chronic inflammation, and dysregulated fibrotic signaling as a major contributor to renal injury (Chen et al., 2025; Gong et al., 2025). Consequently, therapeutic strategies capable of simultaneously modulating these interconnected pathways are increasingly considered of interest to slow disease progression and address the limitations of single-target interventions. From a pharmacological perspective, such multi-target modulation is particularly relevant given the complex nature of DN pathophysiology.

### 4.1 Summary of evidence

#### 4.1.1 Renoprotective efficacy and pleiotropic effects of SAL

This meta-analysis quantitatively summarizes the renoprotective effects of SAL in preclinical DN. Across 14 studies, SAL was associated with improvements in primary outcomes, including BG, Scr, BUN, and KI. These benefits were accompanied by reduced inflammatory mediators (IL-1β and TNF-α) and attenuation of oxidative stress (increased SOD activity and decreased MDA levels), together with evidence of attenuated profibrotic signaling, including lower TGF-β1 in studies reporting this outcome. These effects were observed across different experimental models and outcome measures, although substantial between-study heterogeneity was present. Subgroup analyses indicated that higher-dose SAL (≥100 mg/kg) was associated with larger improvements in BG, Scr, and BUN than lower-dose regimens; however, these findings should be interpreted cautiously given the limited number of studies and between-study heterogeneity.

#### 4.1.2 Influence of glycemic control on renal outcomes

Hyperglycemia is a major driver of DN progression, raising the possibility that the observed renoprotective effects of SAL could be secondary to improved glycemic control rather than kidney-specific actions. To examine this, we conducted random-effects study-level meta-regression relating the glucose-lowering effect size to renal outcomes. No clear linear association was detected for Scr (k = 8; P = 0.22) or BUN (k = 6; P = 0.14). Consistently, median-split subgroup analyses by the magnitude of glucose reduction yielded pooled estimates in the same direction across both strata, with no evidence of between-subgroup differences for Scr (Q_b P = 0.37) or BUN (Q_b P = 0.27) (Supplementary Figure S4; Supplementary Table S4). Only two studies reported both urinary protein and blood glucose (k = 2), precluding quantitative assessment of glycemia–proteinuria relationships; these were summarized descriptively (Supplementary Table S4). Sensitivity analyses excluding the single preventive-design study and studies using a lower diabetes-induction threshold did not materially alter the direction of pooled estimates for BG, Scr, BUN, or KI (Supplementary Table S5). The absence of a clear study-level association should be interpreted cautiously and does not rule out glycemia-mediated effects; instead, it underscores uncertainty and the need for mechanistic experiments.

### 4.2 Heterogeneity and robustness

Substantial heterogeneity was observed across the primary endpoints (BG, Scr., BUN, and KI), with I^2^ exceeding 75% in several pooled analyses. Such variability is common in preclinical meta-analyses and likely reflects both biological diversity among animal models and methodological differences in study design and outcome assessment. In response, subgroup and sensitivity analyses were conducted to explore potential sources of heterogeneity and to evaluate the robustness of the pooled estimates. Although these analyses were informative, heterogeneity generally persisted, suggesting that key effect modifiers were unmeasured, inconsistently reported, or not amenable to study-level stratification. In this context, the overall direction of effects across outcomes is likely more reliable than the exact magnitude of the pooled estimates, which should be interpreted cautiously. This caution is particularly warranted for outcomes in which effect estimates were sensitive to analytic restrictions or individual studies, indicating reduced certainty under specific model contexts. Future preclinical studies would benefit from more complete and standardized reporting of model induction criteria, baseline disease severity, intervention timing, assay methods, and core risk-of-bias safeguards, which would help reduce unexplained heterogeneity and strengthen translational inference.

#### 4.2.1 Potential sources of heterogeneity

Prespecified sources included animal species, SAL dose, DN modeling strategies, and treatment duration. SAL exposure ranged from 20 to 200 mg/kg and treatment duration from 4 to 12 weeks, which may contribute to differences in effect magnitude. Species and strain differences may also modify treatment responsiveness. These strains may differ in metabolic trajectory, susceptibility to renal injury, and pharmacodynamic responsiveness. Model-dependent differences in disease induction and baseline disease severity may further contribute to variability in metabolic status and renal injury progression. Between-study variability in model success criteria and the definition of diabetes can shift baseline disease severity and the stage of injury at treatment initiation, thereby increasing heterogeneity. Residual heterogeneity may also arise from variability in how outcomes were measured and reported, including differences in urinary protein measurement type and variation in biological matrices, assay platforms, and sampling time points for key biomarkers, which were inconsistently reported.

#### 4.2.2 Robustness of findings

Despite variability in effect magnitudes, the direction of treatment effects was generally aligned across studies. Across the primary renal outcomes, SAL was consistently associated with lower Scr (n = 13), BUN (n = 12), and KI (n = 8), and no study showed effects clearly favoring control. Leave-one-out sensitivity analyses showed that the pooled estimates were not driven by any single study, as sequential exclusion did not materially change the direction or statistical significance of the primary outcomes (Supplementary Figures S1, S2). Robustness checks, including influence diagnostics and sensitivity analyses addressing small-sample comparisons, supported the stability of the overall interpretation and suggested that some extreme SMDs may be inflated by very small within-group variability. Complementary analyses using mean differences, where units were harmonized, provided a more interpretable scale and were broadly concordant with the standardized results; heterogeneity nevertheless remained substantial, consistent with systematic between-study differences. Overall, while pooled effect sizes should be interpreted cautiously given between-study heterogeneity and study-level methodological limitations, the overall pattern supports a reproducible direction of renoprotection with SAL in preclinical DN models.

### 4.3 Mechanistic interpretation

Integrating our pooled quantitative findings with mechanistic evidence from the included studies, SAL may exert renoprotective effects in preclinical DN through effects on multiple pathogenic pathways. Across inflammatory markers, IL-1β was consistently lower with SAL, whereas the pooled effect for TNF-α did not reach statistical significance. SAL also showed attenuated oxidative stress, as reflected by increased SOD activity and decreased MDA levels. For fibrotic signaling, pooled evidence was heterogeneous, but TGF-β1 measured by Western blot generally trended lower with SAL, which is compatible with partial suppression of fibrotic activation. These findings provide a framework for examining how SAL may influence several interconnected pathogenic pathways in DN, which are discussed in the following sections.

#### 4.3.1 Anti-inflammatory effects

Chronic low-grade inflammation is a well-recognized feature of DN and contributes to tubular injury and interstitial fibrosis (Navarro-González et al., 2011). Consequently, modulation of dysregulated inflammatory signaling has been widely considered a potential strategy for slowing disease progression.

CCL2-mediated recruitment of monocytes/macrophages via CCR2 contributes to persistent renal inflammation and fibrotic remodeling (Guo et al., 2024). In one included study, SAL treatment reduced the expression of CCL2 and CCR2 at both the mRNA and protein levels, while simultaneously increasing the anti-inflammatory cytokine IL-10 and decreasing pro-inflammatory mediators such as TNF-α and IL-6 (An et al., 2025). These molecular changes were accompanied by improvements in tubular injury and glomerular pathological alterations, suggesting that modulation of the CCL2/CCR2 signaling axis may contribute to the anti-inflammatory effects of SAL in DN models.

Several included studies also reported that SAL suppressed activation of the NLRP3 inflammasome pathway, with reduced NLRP3 expression, decreased caspase-1 activation, attenuated gasdermin D (GSDMD) cleavage, and lower levels of the downstream inflammatory cytokines IL-1β and IL-18 (Li et al., 2024; Qin et al., 2025). These changes were accompanied by improved renal histopathology across DN models. Because reactive oxygen species (ROS) is an upstream trigger for inflammasome activation, SAL’s anti-inflammatory effects may partly arise from upstream modulation of cellular redox balance (Tschopp and Schroder, 2010). Iron-related endpoints were rarely assessed, so the contribution of iron-catalyzed oxidative injury to inflammatory amplification, as well as the potential influence of SAL on this axis, remains uncertain.

Overall, the mechanistic evidence suggests that SAL may attenuate DN-associated inflammation through multiple pathways, including CCL2/CCR2 signaling and inhibition of NLRP3 inflammasome–associated pyroptotic responses. This interpretation is consistent with the pooled reduction in IL-1β observed in our meta-analysis. Even so, direct causal relationships remain to be established, and future studies employing pathway-specific inhibition or rescue approaches will be necessary to clarify the mechanistic hierarchy underlying SAL-mediated anti-inflammatory effects.

#### 4.3.2 Antioxidative stress effects

Oxidative stress is a key pathogenic driver in DN initiation and progression (Forbes et al., 2008). Sustained hyperglycemia promotes excessive ROS generation, thereby exacerbating lipid peroxidation, mitochondrial dysfunction, and renal cellular injury. While mitochondrial ROS production is a major contributor, systems-level frameworks also implicate redox-active (“labile”) iron in catalyzing radical formation and lipid peroxidation. This broader perspective highlights that antioxidative interventions may achieve greater efficacy when combined with strategies that limit reactive iron pools (Kell, 2009).

Mechanistic evidence from the included studies suggests that SAL may enhance endogenous antioxidant defenses through activation of redox-related signaling pathways. The Keap1/Nrf2/ARE pathway represents a major antioxidant defense system involved in maintaining redox homeostasis (Tanase et al., 2022). In one included study, SAL was reported to activate Nrf2/ARE signaling and upregulate γ-glutamylcysteine synthetase (γ-GCS), a key enzyme involved in glutathione synthesis, supporting a potential role for Nrf2-dependent transcriptional regulation in mediating SAL-associated attenuation of oxidative stress (Zhang, 2014). However, the upstream regulatory mechanisms of this pathway were not consistently verified across studies and therefore warrant further mechanistic clarification.

Across included studies, SAL was also linked to the reinforcement of mitochondrial stress-adaptation signaling. Other studies implicated pathways associated with mitochondrial resilience, including Akt/GSK-3β signaling (accompanied by higher SOD activity and lower MDA levels) and AMPK/SIRT1/PGC-1α signaling (Piao et al., 2017; Xue et al., 2019; Shati, 2020; Pei et al., 2022).

An unresolved question is whether SAL primarily amplifies antioxidant capacity, for example, by enhancing SOD-related defenses, or also modulates iron-dependent redox chemistry in DN. Experimental studies in non-DN contexts have reported that SAL attenuates iron overload and iron deposition while modulating iron metabolism–related proteins, changes that occur alongside reductions in lipid peroxidation and ferroptosis-related alterations (Yang et al., 2023). However, comparable mechanistic evidence in DN models remains limited. Including direct measurements of labile iron and iron-handling markers would help determine whether SAL’s renoprotective effects reflect antioxidant pathway activation alone or also involve limiting iron-catalyzed oxidative injury. Clarifying this distinction may inform whether therapeutic strategies targeting both ROS production and reactive iron pools provide additional benefit.

#### 4.3.3 Anti-fibrotic effects

Renal fibrosis and glomerulosclerosis constitute the principal structural basis for progressive and ultimately irreversible renal function decline in DN. Among profibrotic mediators, TGF-β1 is widely recognized as a central driver of renal fibrogenesis by promoting extracellular matrix (ECM) production and tissue remodeling (Meng et al., 2016). The TGF-β1/Smad pathway is a core profibrotic axis involved in the initiation and maintenance of renal fibrogenesis in DN.

In the present meta-analysis, the evidence regarding the influence of salidroside (SAL) on TGF-β1 expression was not entirely consistent. The primary pooled estimate did not reach statistical significance and was accompanied by substantial between-study heterogeneity. Nevertheless, sensitivity analyses and subgroup analyses focusing on protein-based measurements suggested a directional association between SAL treatment and lower TGF-β1 levels, indicating that the antifibrotic signal may be sensitive to methodological factors such as assay platform or tissue source.

Mechanistic observations from several included studies provide qualitative support for this interpretation. For example, SAL was reported to suppress renal TGF-β1 expression and attenuate Smad phosphorylation, changes that were accompanied by reduced deposition of fibrosis-related matrix proteins including collagen I and fibronectin (Leng et al., 2019; Qi et al., 2021).

Beyond the TGF-β1/Smad axis, SAL has also been reported to interact with other signaling cascades involved in fibrogenic progression, including Wnt/β-catenin signaling and the AGEs-RAGE/JAK1/STAT3 pathway (Shati and Alfaifi, 2020; Leng et al., 2024). At the histological level, these molecular alterations were often accompanied by decreased myofibroblast activation and reduced extracellular matrix accumulation (Xue et al., 2019; Leng et al., 2024).

Despite these observations, the antifibrotic role of SAL in experimental DN should be interpreted cautiously. The absence of a robust overall effect for TGF-β1 in the pooled analysis suggests that the observed antifibrotic signal may be influenced by experimental context, detection methodology, or model characteristics. While the subgroup findings and mechanistic studies are suggestive, they do not yet establish TGF-β1 modulation as a primary mechanism of SAL-mediated renoprotection. Future studies incorporating pathway-specific inhibition or rescue strategies will be necessary to determine whether reductions in TGF-β1 represent a direct antifibrotic action of SAL or arise secondarily from upstream improvements in metabolic or oxidative stress conditions.

#### 4.3.4 Mechanistic integration

**FIGURE 7:**
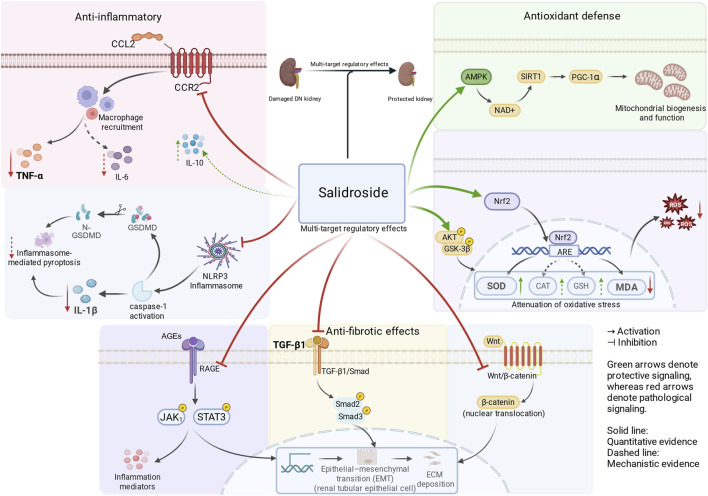
Schematic representation of the potential therapeutic mechanisms of salidroside in diabetic nephropathy. Abbreviations: AMPK, AMP-activated protein kinase; CCL2, C-C motif chemokine ligand 2; EMT, epithelial–mesenchymal transition; JAK, Janus kinase; NLRP3, NLR family pyrin domain containing 3; Nrf2, nuclear factor erythroid 2-related factor 2; SIRT1, sirtuin 1; SOD, superoxide dismutase; TGF-β1, transforming growth factor-beta 1; TNF-α, tumor necrosis factor-alpha.

Oxidative stress triggered by chronic hyperglycemia is widely regarded as an upstream driver of DN progression and may link metabolic disturbance with inflammatory and fibrotic injury in the diabetic kidney (Wang and Zhang, 2024a). Available evidence suggests that SAL may attenuate ROS accumulation and preserve mitochondrial function, thereby contributing to improved cellular redox balance. Such modulation of oxidative stress may subsequently dampen inflammatory pathways, including NLRP3 inflammasome activation and AGEs-RAGE-associated signaling, which are involved in DN-associated renal injury. These changes may in turn reduce profibrotic mediators such as TGF-β1 and limit extracellular matrix deposition (Liu et al., 2023). In this context, the findings of our meta-analysis support a multi-pathway renoprotective profile of SAL in experimental DN. A schematic overview of the proposed mechanistic network is presented in Figure 7.

### 4.4 Translational considerations and limitations

This meta-analysis consolidates directionally consistent preclinical signals indicating that SAL attenuates DN–related phenotypes. However, the current evidence remains hypothesis-generating. Substantial heterogeneity across models, together with the limited number of studies informing several mechanistic biomarkers, limits the strength of causal inference. Translation therefore hinges on whether the proposed mitochondrial–inflammatory–redox framework can be linked to clinically measurable readouts and tested under clinically relevant treatment contexts.

#### 4.4.1 Patient selection and biological heterogeneity

DN is biologically heterogeneous rather than a single entity. Multi-omics and machine-learning approaches have suggested biologically informed sub-phenotypes, including profiles marked by inflammatory activation and impaired mitochondrial fatty-acid oxidation (Kang et al., 2015; Reznichenko et al., 2024; Lin et al., 2025). The preclinical findings summarized here are broadly consistent with this axis, yet the mechanistic dataset is limited and not suited to patient-level inference. If clinical studies are contemplated, non-stratified enrollment may be inefficient. A pragmatic step is to embed exploratory biomarker-informed stratification from the outset. Circulating metabolite patterns, including acylcarnitines and amino-acid profiles, may provide a feasible approach to test whether a metabolic–inflammatory biotype shows greater responsiveness to SAL.

#### 4.4.2 Dose translation, exposure feasibility, and polypharmacy

The animal-study literature spans a wide dose range but rarely defines exposure–response relationships. Human dose planning should not rely on allometric scaling alone. Oral bioavailability, clearance, and formulation-dependent exposure need to be characterized to define achievable systemic and renal exposure with acceptable safety margins. Clinical relevance also depends on background therapy. Contemporary DN management is combination-based, whereas SAL has been evaluated largely as monotherapy in animal models. Before efficacy-oriented clinical conclusions are considered, SAL should be tested under standard-of-care conditions, with explicit assessment of pharmacodynamic additivity and herb–drug interaction liability, including potential effects on drug-metabolizing enzymes and transporters.

#### 4.4.3 Translatable endpoints and monitoring

Most preclinical studies rely on terminal histology and short-term biochemical markers, limiting clinical monitoring. Translation would benefit from endpoints that support longitudinal assessment and patient-facing interpretability. Translational imaging advances, including hyperpolarized ^13^C-pyruvate MRI in clinical kidney settings, can quantify metabolic reprogramming through lactate-to-pyruvate flux (Liu et al., 2025). In future translational studies of SAL, integrating lactate/pyruvate flux with complementary proton MRI parameters could provide a practical bridge between mechanistic hypotheses and clinical monitoring. These approaches remain hypothesis-generating until supported by targeted intervention or rescue designs. At present, these approaches should be viewed as exploratory and require confirmation in studies incorporating targeted intervention or rescue designs.

### 4.5 Methodological constraints and risk of bias

Several limitations should be considered. First, some outcomes were not reported numerically and were extracted from graphs, which may introduce measurement error and information bias. Second, substantial heterogeneity was present across studies in SAL dose (20–200 mg/kg), treatment duration (4–12 weeks), and DN modeling strategies. Different models may reflect distinct disease stages and severity and thereby affect renal and metabolic endpoints. Heterogeneity remained high for key outcomes (BG, Scr, BUN, and KI) despite subgroup analyses according to species, dose, duration, and modeling approach, suggesting additional variability related to experimental design, assay methods, sampling time points, and limited sample sizes. Third, most studies used male animals, limiting generalizability and potentially obscuring sex-specific efficacy or toxicity; future work should include females and report sex-stratified outcomes. Fourth, SYRCLE assessments suggested predominantly unclear risk of bias, especially for allocation concealment and blinding, consistent with incomplete methodological reporting; future studies should adhere to ARRIVE 2.0 and consider prospective preregistration to improve transparency and internal validity. Finally, unusually large standardized mean differences in some outcomes may reflect low within-group variability, because small standard deviations can inflate standardized effects; pooled magnitudes were therefore interpreted cautiously and examined in sensitivity analyses.

#### 4.5.1 Impact of bias and potential overestimation of effects

In addition to these constraints, risk of bias and publication bias may have contributed to an overestimation of treatment effects. Incomplete reporting of random sequence generation, allocation concealment, and blinding is associated with inflated effect sizes in preclinical research, particularly for laboratory outcomes, and may therefore have influenced pooled magnitudes. Moreover, visual funnel plot asymmetry and, where applicable (k ≥ 10), Begg’s and Egger’s tests for Scr and BUN suggested potential small-study effects, raising the possibility that studies reporting larger benefits of SAL were more likely to be published or identified. Although trim-and-fill analyses under a random-effects model using REML did not impute missing studies for Scr or BUN and did not materially change pooled estimates, these methods have limited sensitivity under substantial heterogeneity and when the number of studies is modest; therefore, publication bias cannot be ruled out. Overall, while effect directions were generally consistent, the magnitude of pooled effects should be interpreted cautiously.

## 5 Conclusion

Current preclinical evidence, quantitatively synthesized in this systematic review, supports the renoprotective potential of SAL in DN. Despite substantial heterogeneity across animal models, the directional consistency of the findings suggests that SAL may ameliorate renal dysfunction and improve metabolic disturbances. However, the evidence supporting structural or histopathological improvement is comparatively limited and should therefore be interpreted with caution. Mechanistically, unlike single-target interventions, SAL may act through multiple pathways, encompassing oxidative stress defense (Nrf2/ARE axis), attenuating NLRP3 inflammasome activation and NF-κB signaling, and fibrosis inhibition (TGF-β1/Smad pathway). Notably, given the heterogeneity across models and the limited number of studies for several biomarker outcomes, these mechanistic signals should be regarded as hypothesis-generating rather than evidence of a defined causal pathway. This multi-target activity may help interrupt the oxidative–inflammatory–fibrotic cascade that drives DN progression. In summary, although the translational relevance of these findings is constrained by interspecies variability and the absence of standardized dosing regimens, the evidence remains hypothesis-generating and may be influenced by methodological limitations, including unclear risk of bias and potential small-study effects. The present evidence therefore supports SAL as a promising multi-target bioactive candidate. Future investigations should use rigorous experimental designs with ARRIVE-aligned reporting and prospective protocol preregistration, to delineate the hierarchical relationships among these molecular pathways and to systematically characterize the pharmacokinetic properties of SAL, thereby enabling a more informed evaluation of its therapeutic potential.

## Data Availability

The original contributions presented in the study are included in the article/Supplementary Material, further inquiries can be directed to the corresponding author.
