# Supplementary material for: Anti-inflammatory and antioxidant effects of salidroside in diabetic nephropathy: a systematic review and meta-analysis of preclinical studies
Source: Front Pharmacol. 2026 Apr 10;17:1793037. doi: 10.3389/fphar.2026.1793037 (PMC13106185; doi:10.3389/fphar.2026.1793037)
Supplement: Supplementary file 1 [file Supplementaryfile1.docx]

Supplementary Material

**Supplementary Table S1.** Detailed search strategies used for each database.

| **Database** | **Search strategy** | | |
| --- | --- | --- | --- |
| **PubMed** | ((Salidroside[Title/Abstract] OR Rhodioloside[Title/Abstract] OR Rhodiola[Title/Abstract] OR Rhodosin[Title/Abstract]) AND (animal*[Title/Abstract] OR rat[Title/Abstract] OR rats[Title/Abstract] OR mouse[Title/Abstract] OR mice[Title/Abstract] OR "animal model"[Title/Abstract] OR "experimental study"[Title/Abstract])) AND (("Diabetic Nephropathies"[Mesh]) OR (Nephropathies, Diabetic[Title/Abstract] OR Nephropathy, Diabetic[Title/Abstract] OR Diabetic Nephropathy[Title/Abstract] OR Diabetic Nephropathies[Title/Abstract] OR Diabetic Kidney Disease[Title/Abstract] OR Diabetic Kidney Diseases[Title/Abstract] OR Kidney Disease, Diabetic[Title/Abstract] OR Kidney Diseases, Diabetic[Title/Abstract] OR Diabetic Glomerulosclerosis[Title/Abstract] OR Glomerulosclerosis, Diabetic[Title/Abstract] OR Intracapillary Glomerulosclerosis[Title/Abstract] OR Nodular Glomerulosclerosis[Title/Abstract] OR Kimmelstiel-Wilson Disease[Title/Abstract] OR Kimmelstiel Wilson Disease[Title/Abstract] OR Kimmelstiel-Wilson Syndrome[Title/Abstract] OR Syndrome, Kimmelstiel-Wilson[Title/Abstract] OR Diabetic Renal Disease[Title/Abstract] OR Diabetic Renal Injury[Title/Abstract] OR Diabetic Renal Fibrosis[Title/Abstract] OR Diabetic Renal Dysfunction[Title/Abstract] OR Diabetic Kidney Injury[Title/Abstract] OR DKD[Title/Abstract] OR DN[Title/Abstract])) | | |
| **Cochrane** | **#** | Terms |  |
|  | **1** | MeSH descriptor: [Diabetic Nephropathies] explode all trees |  |
|  | **2** | (Nephropathies, Diabetic):ti,ab,kw OR (Nephropathy, Diabetic):ti,ab,kw OR (Diabetic Nephropathy):ti,ab,kw OR (Diabetic Nephropathies):ti,ab,kw OR (Diabetic Kidney Disease):ti,ab,kw OR (Diabetic Kidney Diseases):ti,ab,kw OR (Kidney Disease, Diabetic):ti,ab,kw OR (Kidney Diseases, Diabetic):ti,ab,kw OR (Diabetic Glomerulosclerosis):ti,ab,kw OR (Glomerulosclerosis, Diabetic):ti,ab,kw OR (Intracapillary Glomerulosclerosis):ti,ab,kw OR (Nodular Glomerulosclerosis):ti,ab,kw OR (Kimmelstiel-Wilson Disease):ti,ab,kw OR (Kimmelstiel Wilson Disease):ti,ab,kw OR (Kimmelstiel-Wilson Syndrome):ti,ab,kw OR (Syndrome, Kimmelstiel-Wilson):ti,ab,kw OR (Diabetic Renal Disease):ti,ab,kw OR (Diabetic Renal Injury):ti,ab,kw OR (Diabetic Renal Fibrosis):ti,ab,kw OR (Diabetic Renal Dysfunction):ti,ab,kw OR (Diabetic Kidney Injury):ti,ab,kw OR (DKD):ti,ab,kw OR (DN):ti,ab,kw |  |
|  | **3** | #1 OR #2 |  |
|  | **4** | (Salidroside):ti,ab,kw OR (Rhodioloside):ti,ab,kw OR (Rhodiola):ti,ab,kw OR (Rhodosin):ti,ab,kw |  |
|  | **5** | #4 and #3 |  |
|  | **6** | (animal):ti,ab,kw OR (rat):ti,ab,kw OR (rats):ti,ab,kw OR (mouse):ti,ab,kw OR (mice):ti,ab,kw OR (animal model):ti,ab,kw OR (experimental study):ti,ab,kw |  |
|  | **7** | #5 and #6 |  |
| **Embase** | **#** | Terms |  |
|  | **1** | 'diabetic nephropathy'/exp |  |
|  | **2** | Nephropathies:ti,ab,kw AND diabetic:ti,ab,kw OR (nephropathy:ti,ab,kw AND diabetic:ti,ab,kw) OR (diabetic:ti,ab,kw AND nephropathy:ti,ab,kw) OR (diabetic:ti,ab,kw AND nephropathies:ti,ab,kw) OR (diabetic:ti,ab,kw AND kidney:ti,ab,kw AND disease:ti,ab,kw) OR (diabetic:ti,ab,kw AND kidney:ti,ab,kw AND diseases:ti,ab,kw) OR (kidney:ti,ab,kw AND disease:ti,ab,kw AND diabetic:ti,ab,kw) OR (kidney:ti,ab,kw AND diseases:ti,ab,kw AND diabetic:ti,ab,kw) OR (diabetic:ti,ab,kw AND glomerulosclerosis:ti,ab,kw) OR (glomerulosclerosis:ti,ab,kw AND diabetic:ti,ab,kw) OR (intracapillary:ti,ab,kw AND glomerulosclerosis:ti,ab,kw) OR (nodular:ti,ab,kw AND glomerulosclerosis:ti,ab,kw) OR ('kimmelstiel wilson':ti,ab,kw AND disease:ti,ab,kw) OR (kimmelstiel:ti,ab,kw AND wilson:ti,ab,kw AND disease:ti,ab,kw) OR ('kimmelstiel wilson':ti,ab,kw AND syndrome:ti,ab,kw) OR (syndrome:ti,ab,kw AND 'kimmelstiel wilson':ti,ab,kw) OR (diabetic:ti,ab,kw AND renal:ti,ab,kw AND disease:ti,ab,kw) OR (diabetic:ti,ab,kw AND renal:ti,ab,kw AND injury:ti,ab,kw) OR (diabetic:ti,ab,kw AND renal:ti,ab,kw AND fibrosis:ti,ab,kw) OR (diabetic:ti,ab,kw AND renal:ti,ab,kw AND dysfunction:ti,ab,kw) OR (diabetic:ti,ab,kw AND kidney:ti,ab,kw AND injury:ti,ab,kw) OR dkd:ti,ab,kw OR dn:ti,ab,kw |  |
|  | **3** | #1 OR #2 |  |
|  | **4** | salidroside:ti,ab,kw OR rhodioloside:ti,ab,kw OR rhodiola:ti,ab,kw OR rhodosin:ti,ab,kw |  |
|  | **5** | animal:ti,ab,kw OR rat:ti,ab,kw OR rats:ti,ab,kw OR mouse:ti,ab,kw OR mice:ti,ab,kw OR 'animal model':ti, ab,kw OR 'experimental study':ti,ab,kw |  |
|  | **6** | #3 AND #4 AND #5 |  |
| **Web of Science** | TS=(Nephropathies, Diabetic OR Nephropathy, Diabetic OR Diabetic Nephropathy OR Diabetic Nephropathies OR Diabetic Kidney Disease OR Diabetic Kidney Diseases OR Kidney Disease, Diabetic OR Kidney Diseases, Diabetic OR Diabetic Glomerulosclerosis OR Glomerulosclerosis, Diabetic OR Intracapillary Glomerulosclerosis OR Nodular Glomerulosclerosis OR Kimmelstiel-Wilson Disease OR Kimmelstiel Wilson Disease OR Kimmelstiel-Wilson Syndrome OR Syndrome, Kimmelstiel-Wilson OR Diabetic Renal Disease OR Diabetic Renal Injury OR Diabetic Renal Fibrosis OR Diabetic Renal Dysfunction OR Diabetic Kidney Injury OR DKD OR DN OR Diabetic Nephropathies) AND TS=(Salidroside OR Rhodioloside OR Rhodiola OR Rhodosin) AND TS=(animal OR rat OR rats OR mouse OR mice OR "animal model" OR "experimental study") | |  |
| **CNKI** | SU='Salidroside OR Rhodioloside OR Rhodioloside OR Salidroside OR Rhodosin OR Rhodiola Extract OR Rhodiola Extract OR Rhodiola rosea Extract OR Rhodiola crenulata Extract' AND SU='Diabetic Nephropathy OR Diabetic Nephropathy OR Diabetic Kidney Injury OR Diabetic Renal Fibrosis OR Diabetic Renal Insufficiency OR Diabetic Renal Impairment OR Diabetic Renal Lesion OR DKD OR DN OR Diabetic Kidney Disease OR Diabetic Renal Impairment OR Diabetic Nephropathy' AND TKA='Animal OR Experiment OR Rat OR Mouse OR Model OR Animal Experiment OR Experimental Study' | |  |
| **CBM** | **#** | Terms |  |
|  | **1** | "Diabetic nephropathy" [unweighted:extended] |  |
|  | **2** | ( "Diabetic Nephropathy"[Common Field:Smart] OR "Diabetic Nephropathy"[Common Field:Smart] OR "Diabetic Kidney Injury"[Common Field:Smart] OR "Diabetic Renal Fibrosis"[Common Field:Smart] OR "Diabetic Renal Insufficiency"[Common Field:Smart] OR "Diabetic Renal Impairment"[Common Field:Smart] OR "Diabetic Renal Lesion"[Common Field:Smart] OR "DKD"[Common Field:Smart] OR "DN"[Common Field:Smart] OR "Diabetic Kidney Disease"[Common Field:Smart] OR "Diabetic Renal Impairment"[Common Field:Smart] OR "Diabetic Nephropathy"[Common Field:Smart] ) |  |
|  | **3** | (#2) OR (#1) |  |
|  | **4** | ( "Salidroside"[Common Field:Smart] OR "Rhodioloside"[Common Field:Smart] OR "Rhodiola Extract"[Common Field:Smart] ) |  |
|  | **5** | ( "Animal"[Common Field:Smart] OR "Experiment"[Common Field:Smart] OR "Rat"[Common Field:Smart] OR "Mouse"[Common Field:Smart] OR "Model"[Common Field:Smart] OR "Animal Experiment"[Common Field:Smart] OR "Experimental Study"[Common Field:Smart] ) |  |
|  | **6** | (#3) AND (#4) AND (#5) |  |
| **WanFang** | Topic:(Salidroside OR Rhodioloside OR Rhodioloside OR Salidroside OR Rhodosin OR Rhodiola Extract OR Rhodiola Extract OR Rhodiola rosea Extract OR Rhodiola crenulata Extract) AND Topic:(Diabetic Nephropathy OR Diabetic Nephropathy OR Diabetic Kidney Injury OR Diabetic Renal Fibrosis OR Diabetic Renal Insufficiency OR Diabetic Renal Impairment OR Diabetic Renal Lesion OR DKD OR DN OR Diabetic Kidney Disease OR Diabetic Renal Impairment OR Diabetic Nephropathy) AND Topic:(Animal OR Experiment OR Rat OR Mouse OR Model OR Animal Experiment OR Experimental Study) | |  |
| **VIP** | ((((((((((Title or Keyword=Salidroside OR Title or Keyword=Rhodioloside) OR Title or Keyword=Rhodioloside) OR Title or Keyword=Salidroside) OR Title or Keyword=Rhodosin) OR Title or Keyword=Rhodiola Extract) OR Title or Keyword=Rhodiola Extract) OR Title or Keyword=Rhodiola rosea Extract) OR Title or Keyword=Rhodiola crenulata Extract) AND (((((((((((Title or Keyword=Diabetic Nephropathy OR Title or Keyword=Diabetic Nephropathy) OR Title or Keyword=Diabetic Kidney Injury) OR Title or Keyword=Diabetic Renal Fibrosis) OR Title or Keyword=Diabetic Renal Insufficiency) OR Title or Keyword=Diabetic Renal Impairment) OR Title or Keyword=Diabetic Renal Lesion) OR Title or Keyword=DKD) OR Title or Keyword=DN) OR Title or Keyword=Diabetic Kidney Disease) OR Title or Keyword=Diabetic Renal Impairment) OR Title or Keyword=Diabetic Nephropathy)) AND ((((((Title or Keyword=Animal OR Title or Keyword=Experiment) OR Title or Keyword=Rat) OR Title or Keyword=Mouse) OR Title or Keyword=Model) OR Title or Keyword=Animal Experiment) OR Title or Keyword=Experimental Study)) | |  |

**Supplementary Table S2** SAL source and purity information.

| Study (year) | SAL supplier/source (as reported) | Catalog No. | Purity (as reported) |
| --- | --- | --- | --- |
| An et al., 2025 | Nanjing Saihongrui Biotechnology Co., Ltd. | S817419 | — |
| Guo et al., 2018 | — | — | — |
| Leng et al., 2019 | Sigma | — | ≥98% |
| Leng et al., 2024 | Shanghai Yuanye Bio-Technology Co., Ltd. | — | ≥98% |
| Li et al., 2024 | Beyotime Biotechnology | SM8036 | — |
| Pei et al., 2022 | — | — | — |
| Piao et al., 2017 | Solarbio (Beijing, China) | — | — |
| Qi et al., 2021 | Gerui Kechuang (Beijing, China) | — | >99% |
| Qin et al., 2025 | Dalian Meilun Biotechnology Co., Ltd. | — | >95% |
| Shati and Alfaifi, 2020 (A) | — | — | — |
| Shati, 2020 (B) | Sigma-Aldrich | SMB00072 | — |
| Wu et al., 2016 | National Institute for Food and Drug Control (Beijing, China) | — | ≥98% |
| Xue et al., 2019 | National Institute for Food and Drug Control (Beijing, China) | — | >98% |
| Zhang, 2014 | — | — | — |

**Note.** SAL supplier/source, catalog number, and purity were extracted as reported in the included studies. “—” indicates that the corresponding information was not reported. SAL, salidroside.

**Supplementary Figure S1.** Leave-one-out sensitivity analyses for the primary outcomes.

**
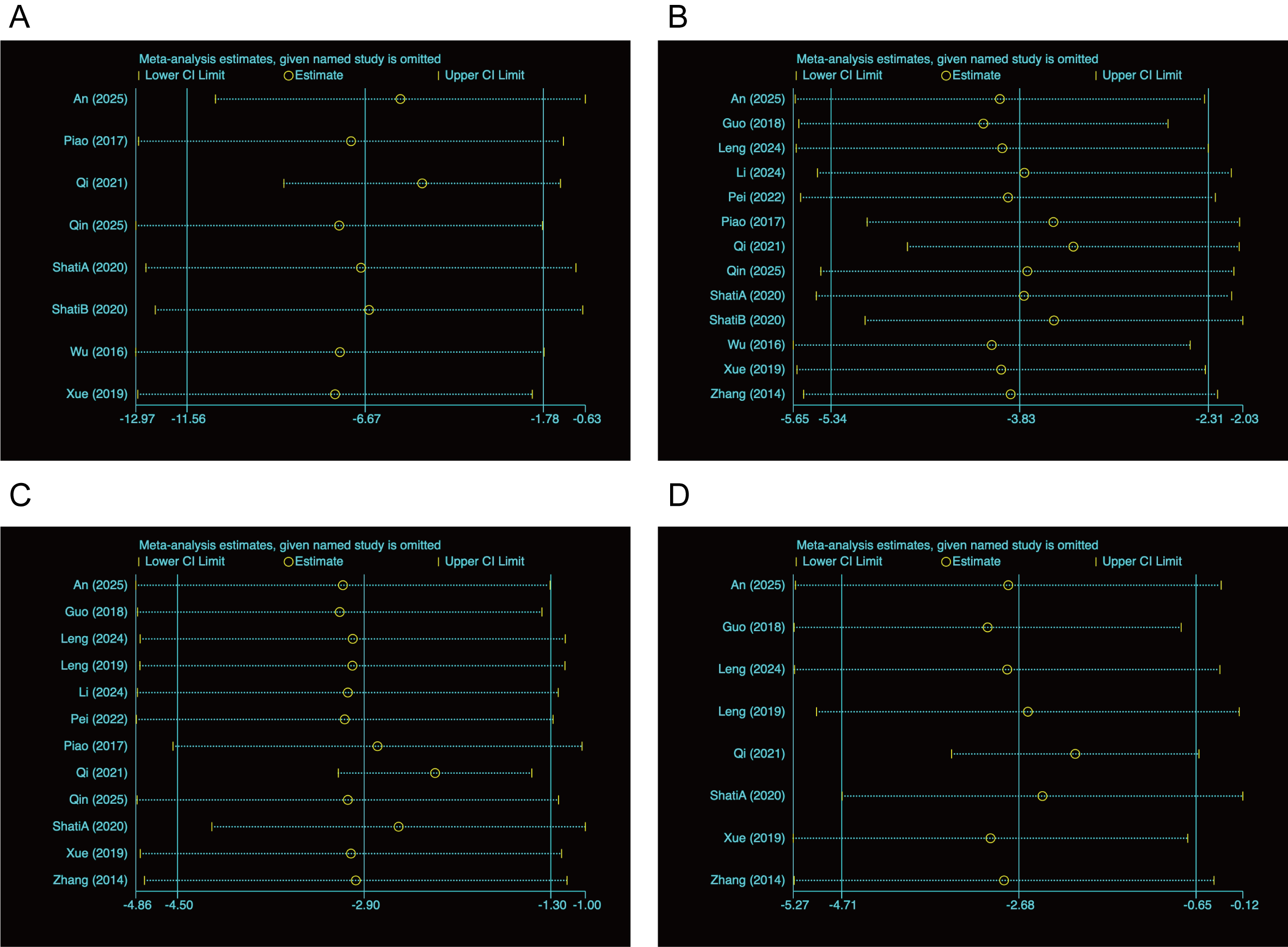
**

**Note.** Leave-one-out meta-analysis estimates are shown after omitting each study in turn for **(A)** blood glucose (BG), **(B)** serum creatinine (Scr), **(C)** blood urea nitrogen (BUN), and **(D)** kidney index (KI). Points indicate the re-estimated pooled effect and horizontal lines represent the corresponding 95% confidence intervals.

**Supplementary Figure S2.** Baujat-type influence diagnostics (leave-one-out) for the primary outcomes.

**
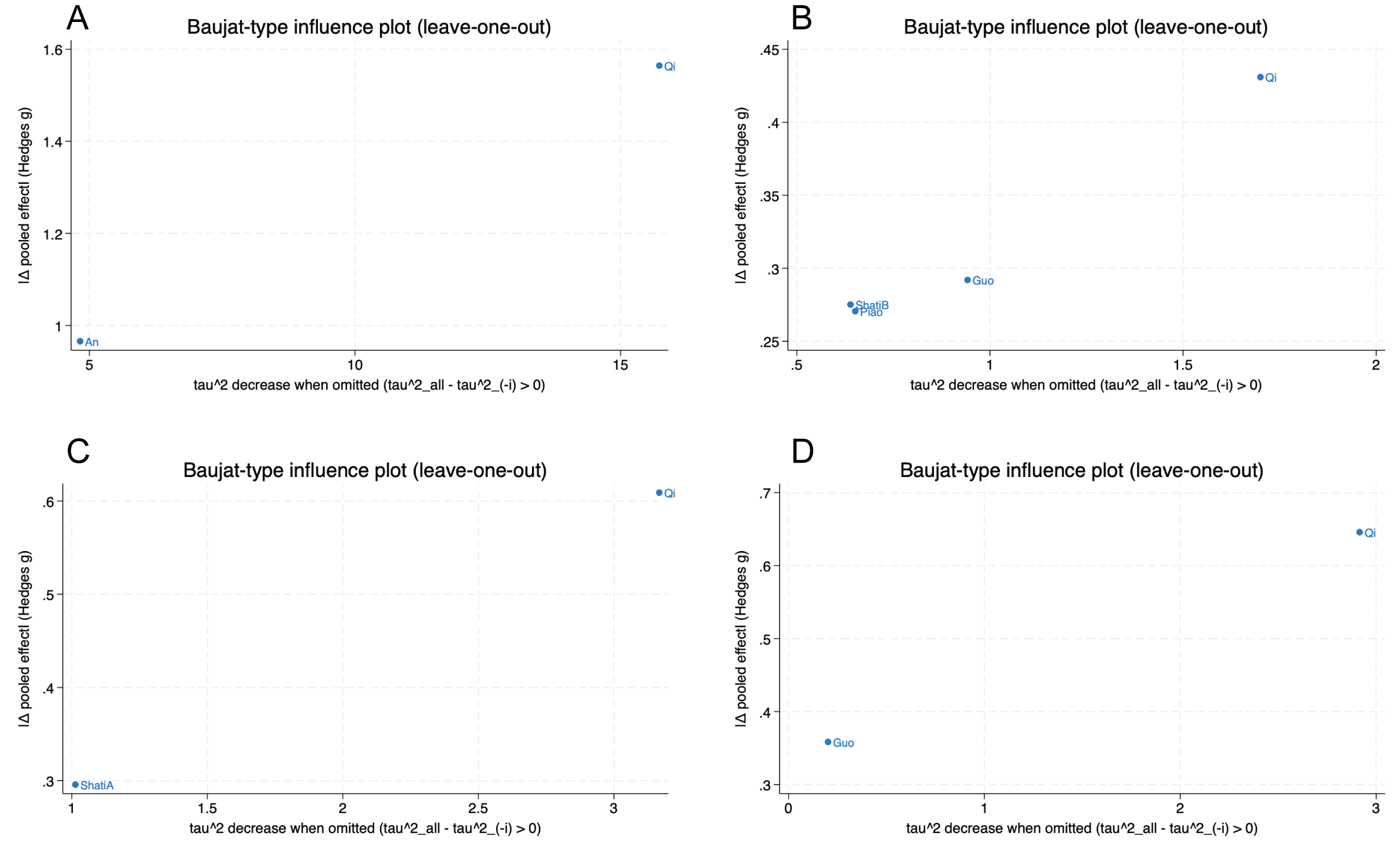
Note.** Baujat-type leave-one-out influence plots are shown for (A) BG, (B) Scr, (C) BUN, and (D) KI. The x-axis shows the reduction in between-study variance when omitting study *i* (τ²_all - τ²_(-i)), and the y-axis shows the absolute change in the pooled effect (|Δ Hedges’ g|). Points toward the upper right indicate studies contributing more to both heterogeneity and influence on the pooled estimate.

**Supplementary Table S3.** Influence diagnostics and leave-one-out sensitivity analyses (random-effects model; REML with HKSJ adjustment)

| Outcome | Main pooled effect | | | Pooled effect after omitting influential studies | | | |
| --- | --- | --- | --- | --- | --- | --- | --- |
|  | I^2^(%) | ***P*** | **Hedges’ g** (95% CI) | Influential studies | I^2^(%) | ***P*** | **Hedges’ g** (95% CI) |
| BG | 90.9% | <0.001 | -6.67 (-11.56, -1.78) | Qi (2021); An (2025) | 84.2% | <0.001 | -3.93 (-6.63, -1.22) |
| Scr | 84.8% | <0.001 | -3.83 (-5.34, -2.31) | Qi (2021) | 81.6% | <0.001 | -3.40 (-4.73, -2.06) |
| BUN | 78.8% | <0.001 | -2.90 (-4.50, -1.30) | Qi (2021) | 61.7% | 0.004 | -2.29 (-3.12, -1.46) |
| KI | 86.5% | <0.001 | -2.68 (-4.71, -0.65) | Qi (2021) | 80.3% | <0.001 | -2.04 (-3.46, -0.62) |

**Note.** Random-effects meta-analyses were performed using REML with Hartung–Knapp–Sidik–Jonkman adjustment. Influential studies were identified via leave-one-out analyses and Baujat plots; pooled effects were recalculated after omitting influential studies. *P* is from Cochran’s Q test for heterogeneity and I² indicates the proportion of total variability due to between-study heterogeneity. ***Abbreviations:*** BG, blood glucose; Scr, serum creatinine; BUN, blood urea nitrogen; KI, kidney index.

**Supplementary Figure S3**. Effect of salidroside on urinary protein outcomes: sensitivity pooling and stratified analysis by reporting metric.


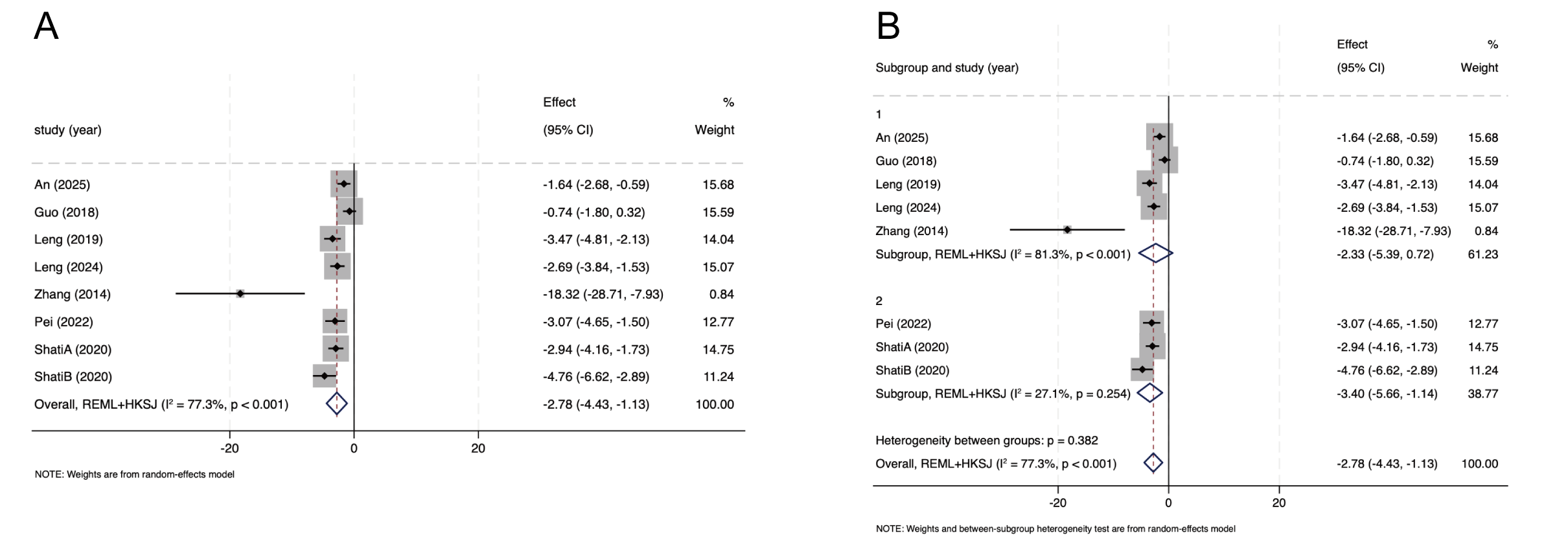


**Note. (A)** Sensitivity meta-analysis pooling all urinary protein (UP) outcomes (24-h excretion, mg/24 h, n = 5; concentration, mg/dL, n = 3; total n = 8) comparing salidroside (SAL) with diabetic nephropathy (DN) model controls. **(B)** Subgroup analysis by reporting metric (1 = mg/24 h, 2 = mg/dL). Effect sizes are Hedges’ g (95% CI; random-effects); negative values indicate lower UP with SAL. I² quantifies heterogeneity.

**Supplementary Figure S4.** Study-level association between blood-glucose effect size and renal outcomes.


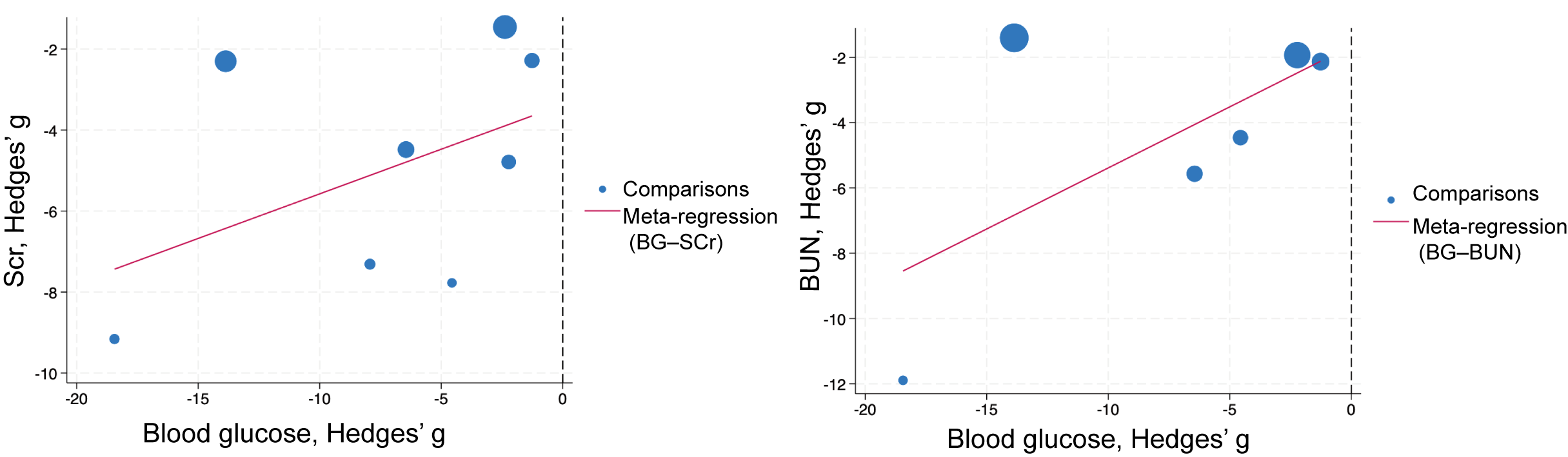


**Note.** Each point represents one study. The x-axis shows the blood-glucose effect (Hedges’ g) and the y-axis shows renal outcomes for (A) Scr and (B) BUN, with effects computed as SAL minus DN model control (more negative values favor SAL). Solid lines indicate fitted study-level random-effects meta-regression (REML); bubble size is proportional to inverse-variance weight (1/SE²). The dashed vertical line denotes no glucose-lowering effect (g_BG = 0).

**Supplementary Table S4**. Meta-regression and glycemic-response subgroup analyses for renal outcomes.

| Outcome | *k* (meta-regression) | Meta-regression, β (REML) | *P* (meta-regression) | Pooled effect (low glycemic response), Hedges’ g (95% CI) | Pooled effect (high glycemic response), Hedges’ g (95% CI) | *P* (between-subgroup difference; Q_b test) | Overall heterogeneity, I² (%) |
| --- | --- | --- | --- | --- | --- | --- | --- |
| Scr | 8 | 0.21 | 0.22 | -3.801 (-6.39, -1.22) | -5.593 (-8.56, -2.63) | 0.37 | 87.9% |
| BUN | 6 | 0.33 | 0.14 | -2.665 (-4.10, -1.23) | -6.061 (-11.91, -0.21) | 0.27 | 92.4% |
| 24-h UP | 2 | — | — | — | — | — | — |

**Note.** Effect sizes are SMDs (Hedges’ g) computed as SAL minus DN model control; negative values indicate lower outcomes in the SAL group. Glycemic-response subgroups were defined using a median split of the blood-glucose effect size (g_BG), where more negative g_BG indicates greater glucose lowering. Meta-regression was performed at the study level. For urinary protein (UP), only two comparisons reported both blood glucose and UP (*k* = 2); therefore, meta-regression and subgroup comparisons were not performed.

**Supplementary Table S5**. Sensitivity analyses of the primary outcomes excluding studies with low diabetes-induction thresholds and excluding the preventive-design study.

| Outcome | Main analysis | | | Excluding low-threshold induction studies | | | Excluding the preventive-design study | | |
| --- | --- | --- | --- | --- | --- | --- | --- | --- | --- |
|  | *k* | Hedges’ g ( 95% CI) | I² (%) | *k* | Hedges’ g ( 95% CI) | I² (%) | *k* | Hedges’ g ( 95% CI) | I² (%) |
| BG | 8 | -6.67 (-11.56, -1.78) | 90.9% | 7 | -5.71(-10.78, -0.63) | 89.8% | 7 | -7.50 (-12.92, -2.08) | 91.1% |
| Scr | 13 | -3.83 (-5.34, -2.31) | 84.8% | 12 | -3.99 (-5.63, -2.34) | 86.0% | 12 | -3.98 (-5.62, -2.33) | 86.0% |
| BUN | 12 | -2.90 (-4.50, -1.30) | 78.8% | 10 | -3.21 (-5.25, -1.16) | 81.7% | 11 | -2.90 (-4.50, -1.30) | 78.8% |
| KI | 8 | -2.68 (-4.71, -0.65) | 86.5% | 6 | -2.98 (-6.01, 0.05) | 90.3% | 7 | -2.68 (-4.71, -0.65) | 86.5% |

**Note.** Values are pooled Hedges’ g (SMD) from random-effects meta-analyses comparing SAL with DN model controls (SAL - control); negative values indicate lower outcomes in the SAL group. “Excluding low-threshold induction studies” removes studies using a lower diabetes-induction threshold (BG ≥ 11.1 mmol/L or fasting blood glucose > 11.1 mmol/L; 2 studies). “Excluding the preventive-design study” removes the preventive SAL study (Xue et al., 2019). *k* denotes the number of comparisons and I² (%) indicates heterogeneity. ***Abbreviations:*** BG, blood glucose; Scr, serum creatinine; BUN, blood urea nitrogen; KI, kidney index.

**Supplementary Figure S5.** Leave-one-out sensitivity analyses for outcomes with very large effects (SMD ≥ 4): IL-1β, SOD, MDA, and TGF-β1.

**
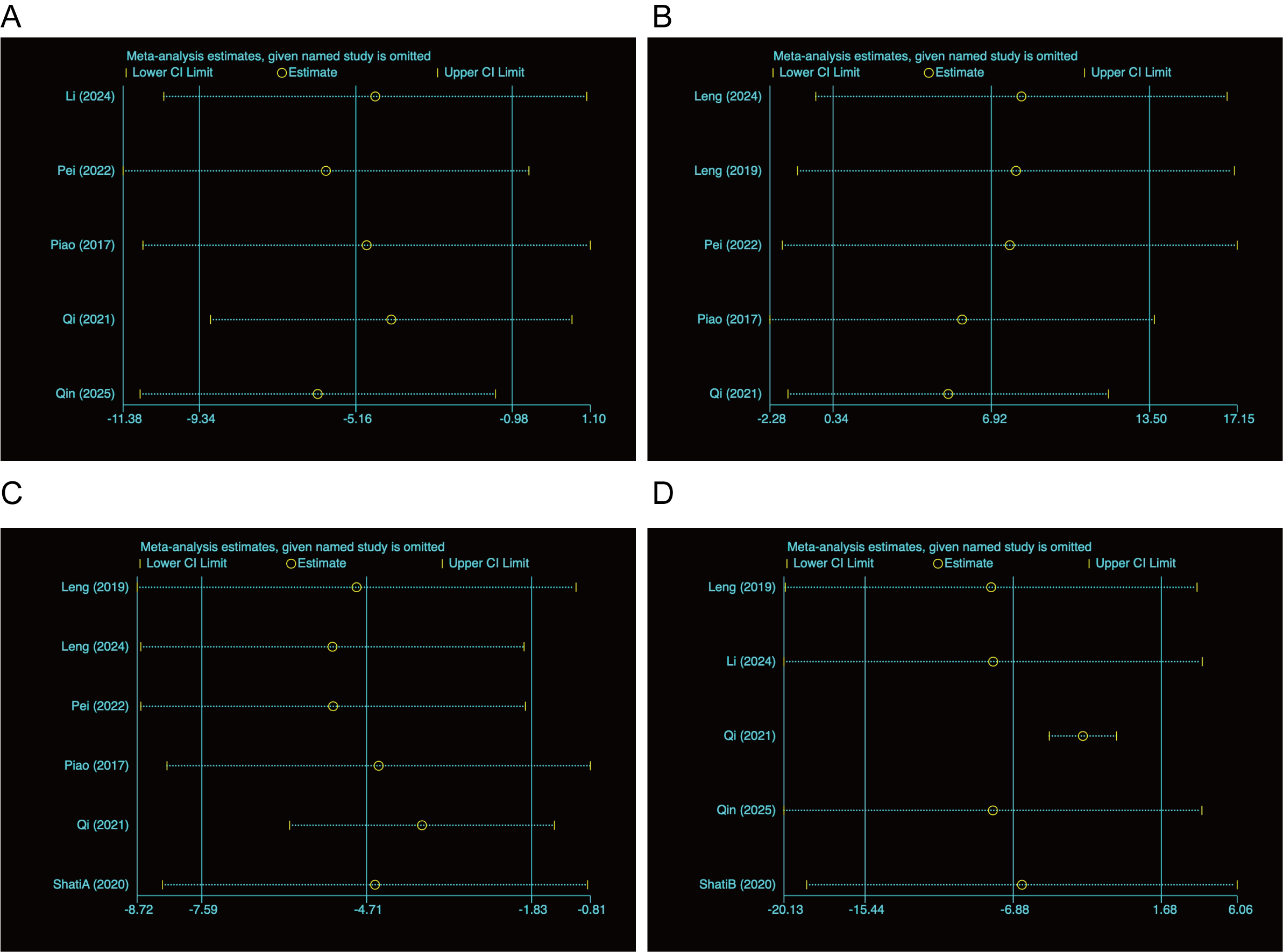
**

**Note.** Leave-one-out meta-analysis estimates are shown after omitting each study in turn for **(A)** interleukin-1β (IL-1β), **(B)** superoxide dismutase (SOD), **(C)** malondialdehyde (MDA), and **(D)** transforming growth factor-β1 (TGF-β1). Points indicate the re-estimated pooled effect and horizontal lines represent the corresponding 95% confidence intervals.

**Supplementary Figure S6.** Baujat-type influence diagnostics (leave-one-out) for outcomes with very large effects (SMD ≥ 4): IL-1β, SOD, MDA, and TGF-β1.

**
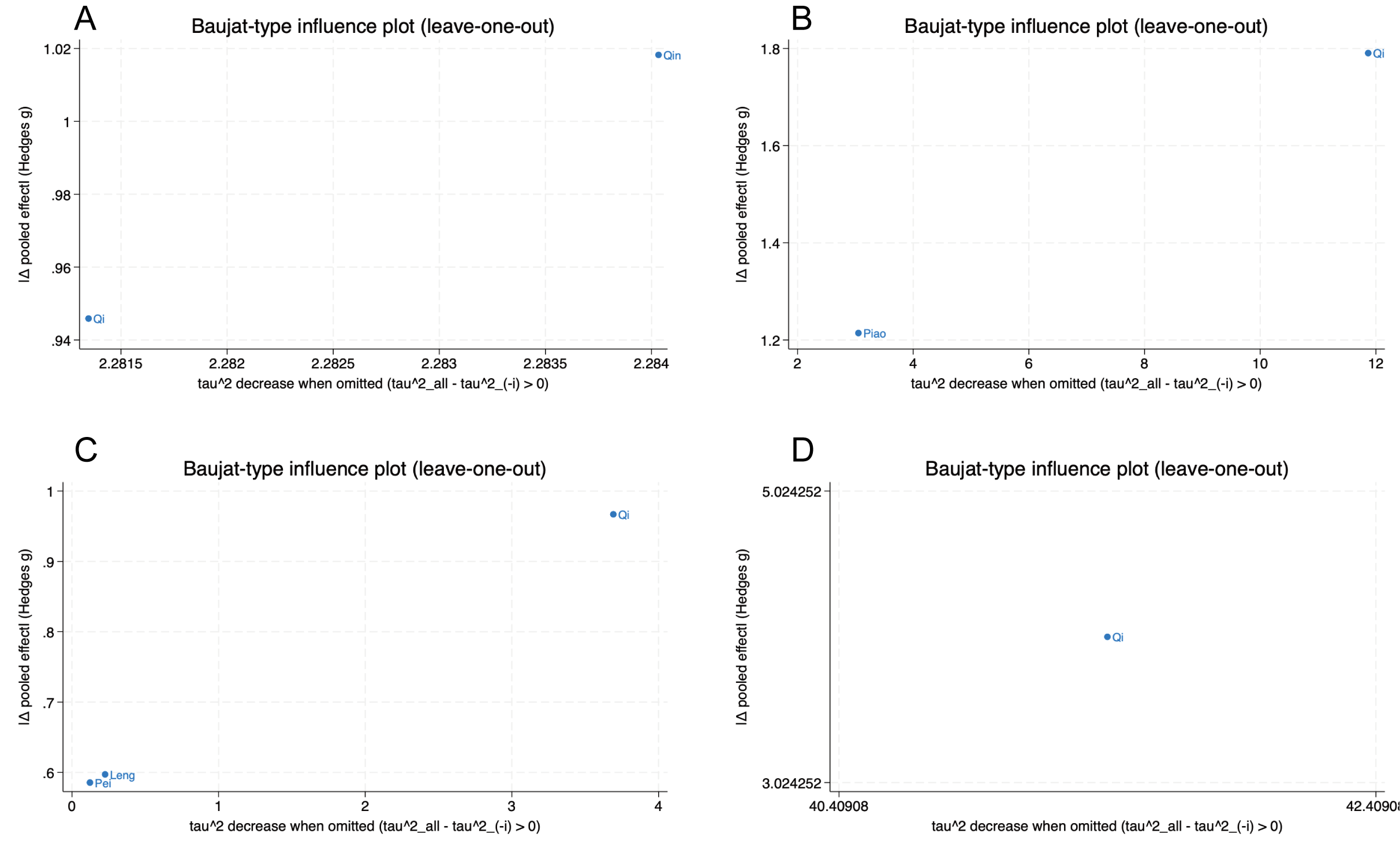
**

**Note.** Baujat-type leave-one-out influence plots are shown for (A) IL-1β, (B) SOD, (C) MDA, and (D) TGF-β1. The x-axis shows the reduction in between-study variance when omitting study *i* (τ²_all - τ²_(-i)), and the y-axis shows the absolute change in the pooled effect (|Δ Hedges’ g|). Points toward the upper right indicate studies contributing more to both heterogeneity and influence on the pooled estimate.

**Supplementary Table S6.** Influence diagnostics and leave-one-out sensitivity analyses (random-effects model; REML with HKSJ adjustment)

| Outcome | Main pooled effect | | | After omitting influential studies | | | |
| --- | --- | --- | --- | --- | --- | --- | --- |
|  | I^2^(%) | ***P*** | **Hedges’ g** (95% CI) | Influential studies | I^2^(%) | ***P*** | **Hedges’ g** (95% CI) |
| IL-1β | 90.1% | <0.001 | -5.16 (-9.34, -0.98) | Qin (2025) | 86.5% | <0.001 | -4.21 (-9.04, 0.61) |
| SOD | 89.2% | <0.001 | 6.92 (0.34, 13.50) | Qi (2021), Piao (2017) | 33.3% | 0.223 | 3.23 (0.93, 5.54) |
| MDA | 84.0% | <0.001 | -4.71 (-7.59, -1.83) | Qi (2021) | 73.2% | 0.005 | -3.75 (-6.06, -1.44) |
| TGF-β1 | 88.9% | <0.001 | -6.88 (-15.44, 1.68) | Qi (2021) | 62.5% | 0.046 | -2.86 (-4.80, -0.92) |

**Note.** Random-effects meta-analyses were performed using REML with Hartung–Knapp–Sidik–Jonkman adjustment. Influential studies were identified using leave-one-out analyses and Baujat diagnostics; pooled estimates were recalculated after omitting influential studies. *P* is from Cochran’s Q test and I² (%) indicates heterogeneity. Effect sizes are Hedges’ g (SMD) computed as SAL minus DN model control. This table summarizes biomarkers with very large pooled effects in the main analysis (SMD ≥ 4). **Abbreviations:** IL-1β, interleukin-1β; SOD, superoxide dismutase; MDA, malondialdehyde; TGF-β1, transforming growth factor-β1.

**Supplementary Table S7.** Stratified meta-analyses of mechanistic biomarkers by sample source and assay type.

| Biomarker | Stratification factor | Subgroup | *k* | I² (%) | SMD (Hedges’ g) (95% CI) |
| --- | --- | --- | --- | --- | --- |
| IL-1β | Sample source | Renal tissue/cortex | 1 | — | — |
|  |  | Serum | 4 | 87.9% | -6.18 (-10.92, -1.43) |
| SOD | Sample source | Renal tissue/cortex | 4 | 91.8% | 7.95 (-1.13, 17.03) |
|  |  | Serum | 1 | — | — |
| MDA | Sample source | Renal tissue/cortex | 5 | 86.6% | -4.89 (-8.72, -1.06) |
|  |  | Serum | 1 | — | — |
| TGF-β1 | Assay level/type | IHC | 1 | — | — |
|  |  | WB | 4 | 62.5% | -2.86 (-4.80, -0.92) |
| TGF-β1 | Assay level/type | WB (sensitivity) | 3 | 0.0% | -2.70 (-3.36, -2.04) |

**Note.** Stratified random-effects meta-analyses were performed by sample source (renal tissue/cortex vs serum) and, where applicable, by assay type (WB vs IHC). Effect sizes are SMDs (Hedges’ g) computed as SAL minus DN model control; negative values indicate lower values in the SAL group. I² (%) indicates between-study heterogeneity. Pooled estimates were not calculated when *k* < 3 and are shown as “—”. “TGF-β1 (sensitivity)” denotes the WB subgroup after excluding the identified outlier. ***Abbreviations:*** WB, Western blot; IHC, immunohistochemistry; IL-1β, interleukin-1β; SOD, superoxide dismutase; MDA, malondialdehyde; TGF-β1, transforming growth factor-β1.

**Supplementary Table S8.** Study-level raw mean differences (MDs) in original units for outcomes with very large pooled effects.

| Outcome | Study (year) | SAL mean ± SD (n) | Control mean ± SD (n) | Raw MD (SAL - control) | SMD (Hedges’ g) |
| --- | --- | --- | --- | --- | --- |
| BG | An (2025) | 21.9 ± 0.44 (10) | 28.7 ± 0.5 (10) | -6.82 | -13.88 |
|  | Piao (2017) | 20.9 ± 0.72 (8) | 26.7 ± 1.44 (8) | -5.49 | -4.56 |
|  | Qi (2021) | 9.9 ± 0.37 (12) | 17.7 ± 0.41 (12) | -7.46 | -18.44 |
|  | Qin (2025) | 14.9 ± 3.01 (10) | 22.7 ± 3.7 (10) | -7.83 | -2.22 |
|  | ShatiA (2020) | 9.9 ± 1.35 (12) | 19.7 ± 1.48 (12) | -9.46 | -6.45 |
|  | ShatiB (2020) | 8.9 ± 0.88 (10) | 18.7 ± 1.58 (10) | -10.59 | -7.93 |
|  | Wu (2016) | 11.9 ± 3.47 (9) | 20.7 ± 3.58 (9) | -8.81 | -2.38 |
|  | Xue (2019) | 18.9 ± 6.52 (5) | 25.7 ± 2.12 (5) | -6.78 | -1.26 |
| IL-1β | Li (2024) | 13.97 ± 2.41 (8) | 40.57 ± 4.01 (8) | -26.6 | -7.6 |
|  | Pei (2022) | 14.92 ± 1.3 (8) | 17.78 ± 0.99 (8) | -2.86 | -2.34 |
|  | Piao (2017) | 14.92 ± 0.46 (8) | 17.78 ± 0.35 (8) | -2.86 | -6.62 |
|  | Qi (2021) | 14.3 ± 1.8 (12) | 38.5 ± 3.2 (12) | -24.2 | -9.00 |
|  | Qin (2025) | 24.37 ± 4.28 (10) | 31 ± 4.45 (10) | -6.63 | -5.16 |
| SOD | Leng (2024) | 50.97 ± 9.40 (10) | 27.08 ± 9.22 (10) | 23.89 | 2.46 |
|  | Leng (2019) | 7.94 ± 1.58 (12) | 3.2 ± 1.02 (12) | 4.74 | 3.44 |
|  | Pei (2022) | 32.87 ± 1.3 (8) | 27.2 ± 1.1 (8) | 5.67 | 4.45 |
|  | Piao (2017) | 32.87 ± 0.46 (8) | 27.2 ± 0.39 (8) | 5.67 | 12.57 |
|  | Qi (2021) | 45.16 ± 2.18 (12) | 15 ± 2.03 (12) | 30.16 | 13.82 |
| MDA | Leng (2019) | 5.84 ± 0.63 (12) | 9.12 ± 0.83 (12) | -3.23 | -4.23 |
|  | Leng (2024) | 8.79 ± 2.7 (10) | 14.14 ± 2.07 (10) | -5.35 | -2.13 |
|  | Pei (2022) | 11.72 ± 0.82 (8) | 13.65 ± 0.88 (8) | -1.93 | -2.15 |
|  | Piao (2017) | 11.72 ± 0.29 (8) | 13.65 ± 0.31 (8) | -1.93 | -6.08 |
|  | Qi (2021) | 4.62 ± 0.5 (12) | 13.07 ± 1.11 (12) | -8.45 | -9.48 |
|  | ShatiA (2020) | 1.26 ± 0.21 (6) | 3.01 ± 0.33 (6) | -1.75 | -5.84 |
| TGF-β1 | Leng (2019) | 1.52 ± 0.19 (12) | 2.1 ± 0.26 (12) | -0.58 | -2.06 |
|  | Li (2024) | 0.4 ±0.09 (8) | 0.71 ± 0.11 (8) | -0.31 | -0.30 |
|  | Qi (2021) | 0.10 ± 0.01 (12) | 0.542 ± 0.03 (12) | -0.44 | -1.98 |
|  | Qin (2025) | 0.61 ± 0.06 (10) | 0.84 ± 0.09 (10) | -0.23 | -3.55 |
|  | ShatiB (2020) | 0.3 ± 0.05 (6) | 0.93 ± 0.07 (6) | -0.63 | -8.09 |

**Note.** Study-level raw MDs (SAL - DN model control) in original units are presented alongside SMDs (Hedges’ g) to aid interpretation of very large standardized effects. Negative values indicate lower values in the SAL group. Raw MDs are intended for within-study interpretation and should not be compared directly across studies due to differences in measurement units and assay methods.

**Supplementary Table S9.** Comparison of pooled mean differences (MDs) and standardized mean differences (SMDs) for the primary outcomes and selected biomarkers with harmonized measurement units across studies.

| Outcome | *k* | Pooled MD | | Pooled SMD | | Unit |
| --- | --- | --- | --- | --- | --- | --- |
|  |  | MD (95% CI) | I^2^ (%) | SMD (Hedges’ g) (95% CI) | I^2^ (%) |  |
| BG | 8 | -7.95 (-9.4, -6.49) | 89.0% | -6.67 (-11.56, -1.78) | 90.9% | mmol/L |
| Scr | 13 | -0.64 (-1.0, -0.29) | 99.0% | -3.83 (-5.34, -2.31) | 84.8% | mg/dL |
| BUN | 12 | -5.73 (-8.71, -2.75) | 94.5% | -2.90 (-4.50, -1.30) | 78.8% | mmol/L |
| KI | 8 | -1.82 (-2.84, -0.80) | 92.0% | -2.68 (-4.71, -0.65) | 86.5% | mg/g |
| IL-1β | 4 | -14.70 (-34.83, 6.69) | 99.5% | -6.18 (-10.92, -1.43) | 87.9% | ng/L |
| SOD | 4 | 16.15 (-4.01, 36.31) | 99.6% | 7.95 (-1.13, 17.03) | 91.8% | U/mg protein |
| MDA | 4 | -4.34 (-9.46, 0.77) | 99.0% | -4.67 (-10.20, 0.87) | 87.8% | nmol/mg protein |
| TGF-β1 | 5 | — | — | -6.88 (-15.44, 1.68) | 88.9% | — |

**Note.** This table compares pooled effects estimated using mean differences (MDs) and standardized mean differences (SMDs; Hedges’ g) for outcomes with harmonized measurement units across studies. For comparability with MDs, SMDs presented in this table were recalculated using the same subset of studies with harmonized units. Consequently, these SMD estimates may differ from those reported in the main analyses, which were based on all available studies regardless of measurement units. Effects were calculated as SAL minus DN model control; negative values indicate lower values in the SAL group. Random-effects meta-analyses were performed using REML with Hartung–Knapp–Sidik–Jonkman adjustment. I² (%) indicates between-study heterogeneity. TGF-β1 was analyzed using SMDs only because MDs could not be harmonized across studies. ***Abbreviations:*** BG, blood glucose; Scr, serum creatinine; BUN, blood urea nitrogen; KI, kidney index; IL-1β, interleukin-1β; SOD, superoxide dismutase; MDA, malondialdehyde; MD, mean difference; SMD, standardized mean difference.

**Supplementary Table S10.** Sensitivity analysis excluding comparisons with very small group sizes (n ≤ 8).

| Outcome | Main analysis | | | Excluding n≤8 | | | Direction changed? (Yes/No) |
| --- | --- | --- | --- | --- | --- | --- | --- |
|  | *k* | **Hedges’ g (95% CI)** | I² (%) | *k* | **Hedges’ g (95% CI)** | I² (%) |  |
| BG | 8 | -6.67 (-11.56, -1.78) | 90.9% | 6 | -8.09 (-14.70, -1.48) | 92.5% | No |
| Scr | 13 | -3.83 (-5.34, -2.31) | 84.8% | 7 | -4.33 (-6.85, -1.81) | 86.2% | No |
| BUN | 12 | -2.90 (-4.50, -1.30) | 78.8% | 6 | -3.96 (-7.88, -0.03) | 87.4% | No |
| KI | 8 | -2.68 (-4.71, -0.65) | 86.5% | 5 | -3.85 (-6.81, -0.9) | 84.3% | No |
| SOD | 5 | 6.92 (0.34, 13.50) | 89.2% | 3 | 6.29 (-9.03, 21.60) | 91.8% | No |
| MDA | 6 | -4.71 (-7.59, -1.83) | 84.0% | 3 | -5.08 (-14.29, 4.13) | 90.6% | No |
| TGF-β1 | 5 | -6.88 (-15.44, 1.68) | 88.9% | 3 | -7.74 (-30.74, 15.25) | 93.1% | No |

**Note.** Random-effects meta-analyses were performed using REML with Hartung–Knapp–Sidik–Jonkman adjustment. “Excluding n ≤ 8” removes comparisons in which ≥1 group had n ≤ 8 animals. “Direction changed” indicates a change in the sign of the pooled estimate versus the main analysis. I² (%) indicates heterogeneity. The IL-1β analysis was not performed because only two comparisons remained after exclusion. ***Abbreviations:*** BG, blood glucose; Scr, serum creatinine; BUN, blood urea nitrogen; KI, kidney index; SOD, superoxide dismutase; MDA, malondialdehyde; TGF-β1, transforming growth factor-β1; SMD, standardized mean difference.

**Supplementary Table S11.** Sensitivity analysis substituting the selected medium-dose salidroside arm with the highest-dose arm in multi-arm studies.

| Sensitivity  analysis | Medium-dose | | | Highest-dose | | |
| --- | --- | --- | --- | --- | --- | --- |
|  | I^2^ | ***P*** | SMD (Hedges’ g) (95% CI) | I^2^ | ***P*** | SMD (Hedges’ g) (95% CI) |
| BG | 90.9% | <0.001 | -6.67 (-11.56, -1.78) | 90.6% | <0.001 | -6.66 (-11.39, -1.92) |
| Scr | 84.8% | <0.001 | -3.83 (-5.34, -2.31) | 83.6% | <0.001 | -3.97 (-5.42, -2.51) |
| BUN | 78.8% | <0.001 | -2.90 (-4.50, -1.30) | 79.5% | <0.001 | -3.09 (-4.68, -1.51) |
| KI | 86.5% | <0.001 | -2.68 (-4.71, -0.65) | 88.8% | <0.001 | -3.10 (-5.24, -0.96) |

**Note.** In multi-arm studies, the main analysis used the pre-specified medium-dose salidroside arm; this sensitivity analysis re-estimated pooled effects after substituting the highest-dose arm from the same studies. Random-effects meta-analyses were performed using REML with Hartung–Knapp–Sidik–Jonkman adjustment. I² (%) indicates between-study heterogeneity and *P* is from Cochran’s Q test. Dose categories were defined as medium-dose (<100 mg/kg) and highest-dose (≥100 mg/kg). ***Abbreviations:*** BG, blood glucose; Scr, serum creatinine; BUN, blood urea nitrogen; KI, kidney index; SMD, standardized mean difference; CI, confidence interval.

**Supplementary Table S12.1.** Subgroup analyses by salidroside dose category.

| Subgroup Outcomes | <100 mg/kg | | | | ≥100 mg/kg | | | | *P* for subgroup differences |
| --- | --- | --- | --- | --- | --- | --- | --- | --- | --- |
|  | *k* | I^2^(%) | ***P*** | SMD (Hedges’ g) (95% CI) | *k* | I^2^(%) | ***P*** | SMD (Hedges’ g) (95% CI) |  |
| BG | 3 | 93.2% | <0.001 | -7.33 (-30.07, 15.42) | 5 | 90.5% | <0.001 | -6.43 (-12.00, -0.87) | 0.874 |
| Scr | 6 | 89.9% | <0.001 | -3.72 (-6.90, -0.54) | 7 | 74.6% | <0.001 | -3.91 (-5.94, -1.89) | 0.896 |
| BUN | 6 | 83.8% | <0.001 | -3.16 (-7.19, 0.87) | 6 | 72.9% | 0.002 | -2.90(-4.51, -1.29) | 0.876 |
| KI | 3 | 93.2% | <0.001 | -3.22 (-13.44, 7.00) | 5 | 79.3% | <0.001 | -2.48 (-4.42, -0.55) | 0.767 |

**Note.** SMDs (Hedges’ g) with 95% CIs are reported. *P* values are from Cochran’s Q test (within subgroups) and the between-subgroup Q test (Q_b; χ²) when estimable. **Abbreviations:** BG, blood glucose; Scr, serum creatinine; BUN, blood urea nitrogen; KI, kidney index; SMD, standardized mean difference; CI, confidence interval. Dose categories were defined as < 100 mg/kg vs ≥ 100 mg/kg.

**Supplementary Table S12.2.** Subgroup analyses by treatment duration.

| Subgroup Outcomes | <10 weeks | | | | ≥10 weeks | | | | *P* for subgroup differences |
| --- | --- | --- | --- | --- | --- | --- | --- | --- | --- |
|  | *k* | I^2^(%) | ***P*** | SMD (Hedges’ g) (95% CI) | *k* | I^2^(%) | ***P*** | SMD (Hedges’ g) (95% CI) |  |
| BG | 3 | 86.3% | <0.001 | -10.50 (-26.25, 5.24) | 5 | 85.6% | <0.001 | -4.42 (-10.38, 1.55) | 0.152 |
| Scr | 6 | 91.4% | <0.001 | -4.61 (-7.86, -1.37) | 7 | 70.0% | 0.003 | -3.09 (-4.80, -1.39) | 0.293 |
| BUN | 5 | 90.9% | <0.001 | -4.11 (-9.56, 1.34) | 7 | 23.6% | 0.249 | -2.21 (-2.91, -1.51) | 0.339 |
| KI | 3 | 95.1% | <0.001 | -4.22 (-13.90, 5.46) | 5 | 62.1% | 0.032 | -1.94 (-3.28, -0.61) | 0.322 |

**Note.** SMDs (Hedges’ g) with 95% CIs are reported. *P* values are from Cochran’s Q test (within subgroups) and the between-subgroup Q test (Q_b; χ²) when estimable. **Abbreviations:** BG, blood glucose; Scr, serum creatinine; BUN, blood urea nitrogen; KI, kidney index; SMD, standardized mean difference; CI, confidence interval. Treatment duration was categorized as <10 weeks vs ≥10 weeks.

**Supplementary Table S12.3.** Subgroup analyses by animal species.

| Subgroup Outcomes | Rats | | | | Mice | | | | *P* for subgroup differences |
| --- | --- | --- | --- | --- | --- | --- | --- | --- | --- |
|  | *k* | I^2^(%) | ***P*** | SMD (Hedges’ g) (95% CI) | *k* | I^2^(%) | ***P*** | SMD (Hedges’ g) (95% CI) |  |
| BG | 5 | 91.2% | <0.001 | -7.45 (-14.81, -0.10) | 3 | 91.5% | <0.001 | -5.51 (-22.46, 11.44) | 0.682 |
| Scr | 9 | 88.5% | <0.001 | -4.73 (-6.81, -2.64) | 4 | 0.0% | 0.539 | -2.10 (-2.94, -1.26) | 0.005 |
| BUN | 9 | 83.7% | <0.001 | -3.37 (-5.72, -1.02) | 3 | 0.0% | 0.391 | -1.92 (-3.35, -0.48) | 0.175 |
| KI | 5 | 91.1% | <0.001 | -3.50 (-7.14, 0.14) | 3 | 51.0% | 0.130 | -1.57 (-3.68, 0.55) | 0.167 |

**Note.** SMDs (Hedges’ g) with 95% CIs are reported. *P* values are from Cochran’s Q test (within subgroups) and the between-subgroup Q test (Q_b; χ²) when estimable. **Abbreviations:** BG, blood glucose; Scr, serum creatinine; BUN, blood urea nitrogen; KI, kidney index; SMD, standardized mean difference; CI, confidence interval. Species were categorized as rats vs mice.

**Supplementary Table S12.4.** Subgroup analyses by DN modeling method.

| Subgroup Outcomes | STZ | | | | HFD+STZ | | | | *P* for subgroup differences |
| --- | --- | --- | --- | --- | --- | --- | --- | --- | --- |
|  | *k* | I^2^(%) | ***P*** | SMD (Hedges’ g) (95% CI) | *k* | I^2^(%) | ***P*** | SMD (Hedges’ g) (95% CI) |  |
| BG | 5 | 86.1% | <0.001 | -9.76 (-16.69, -2.82) | 2 | — | — | — | — |
| Scr | 7 | 81.8% | <0.001 | -5.06 (-7.53, -2.59) | 4 | 55.1% | 0.083 | -3.40 (-5.45, -1.36) | — |
| BUN | 6 | 88.7% | <0.001 | -4.30 (-8.21, -0.40) | 4 | 0.0% | 0.917 | -2.11 (-2.52, -1.70) | — |
| KI | 4 | 87.4% | <0.001 | -3.96 (-8.55, 0.64) | 2 | — | — | — | — |

**Note.** SMDs (Hedges’ g) with 95% CIs are reported. *P* values are from Cochran’s Q test (within subgroups) and the between-subgroup Q test (Q_b; χ²) when estimable. **Abbreviations:** BG, blood glucose; Scr, serum creatinine; BUN, blood urea nitrogen; KI, kidney index; SMD, standardized mean difference; CI, confidence interval; STZ, streptozotocin; HFD, high-fat diet. DN models were categorized as STZ, HFD+STZ, STZ + unilateral nephrectomy, and db/db. Subgroup estimates were calculated only when k ≥ 3; “—” indicates k < 3. The STZ + unilateral nephrectomy and db/db subgroups each included <3 studies across outcomes and were omitted from the table for brevity.

**Supplementary Table S12.5.** Subgroup analyses by animal strains.

| Subgroup Outcomes | SD | | | | Wistar | | | | C57BL/6J | | | |
| --- | --- | --- | --- | --- | --- | --- | --- | --- | --- | --- | --- | --- |
|  | *k* | I^2^(%) | ***P*** | SMD (Hedges’ g) (95% CI) | *k* | I^2^(%) | *P* | SMD (Hedges’ g) (95% CI) | *k* | I^2^(%) | ***P*** | SMD (Hedges’ g) (95% CI) |
| BG | 3 | 93.6% | <0.001 | -8.05 (-29.44, 13.34) | — | — | — | — | 2 | — | — | — |
| Scr | 5 | 69.5% | 0.011 | -5.67 (-8.66, -2.68) | 4 | 91.6% | <0.001 | -3.63 (-8.20, 0.95) | 3 | 0.0% | 0.946 | -1.46 (-2.53, -0.39) |
| BUN | 6 | 83.0% | <0.001 | -3.88 (-7.63, -0.12) | 3 | 88.7% | 0.917 | -2.59 (-8.73,3.54) | 3 | 0.0% | 0.391 | -1.92 (-3.35, -0.48) |
| KI | 3 | 88.4% | <0.001 | -4.29 (-12.36,3.77) | 2 | — | — | — | 3 | 51.0% | 0.130 | -1.57 (-3.68, 0.55) |

**Note.** SMDs (Hedges’ g) with 95% CIs are reported. *P* values are from Cochran’s Q test (within subgroups) and the between-subgroup Q test (Q_b; χ²) when estimable. **Abbreviations:** BG, blood glucose; Scr, serum creatinine; BUN, blood urea nitrogen; KI, kidney index; SMD, standardized mean difference; CI, confidence interval. Subgroup estimates are shown only when *k* ≥ 3; “—” indicates *k* < 3. The C57BLKS/J subgroup included <3 studies across outcomes and was omitted from the table for brevity. Strains: SD, Sprague–Dawley rats; Wistar, Wistar rats; C57BL/6J, C57BL/6J mice.

**Supplementary Table S12.6.** Subgroup analyses by salidroside purity reporting.

| Subgroup Outcomes | Reported purity ≥95% | | | | Purity not reported | | | | *P* for subgroup differences |
| --- | --- | --- | --- | --- | --- | --- | --- | --- | --- |
|  | *k* | I^2^(%) | ***P*** | SMD (Hedges’ g) (95% CI) | *k* | I^2^(%) | ***P*** | SMD (Hedges’ g) (95% CI) |  |
| BG | 4 | 90.7% | <0.001 | -5.68 (-18.37, 7.01) | 4 | 77.1% | <0.001 | -7.71 (-13.65, -1.77) | 0.644 |
| Scr | 5 | 86.1% | <0.001 | -3.84 (-7.53, -0.14) | 8 | 86.0% | <0.001 | -3.86 (-5.89, -1.83) | 0.988 |
| BUN | 5 | 84.1% | <0.001 | -3.85 (-8.88, 1.18) | 7 | 76.2% | <0.001 | -2.47 (-4.03,-0.92) | 0.474 |
| KI | 5 | 91.1% | <0.001 | -3.50 (-7.14, 0.14) | 3 | 51.0% | 0.130 | -1.57 (-3.68, 0.55) | 0.167 |

**Note.** SMDs (Hedges’ g) with 95% CIs are reported. *P* values are from Cochran’s Q test (within subgroups) and the between-subgroup Q test (Q_b; χ²) when estimable. **Abbreviations:** BG, blood glucose; Scr, serum creatinine; BUN, blood urea nitrogen; KI, kidney index; SMD, standardized mean difference; CI, confidence interval. “Reported purity ≥95%” indicates studies reporting salidroside purity; “Purity not reported” indicates studies without explicit purity information.

**Supplementary Figure S7**. Bubble plots of univariable random-effects meta-regression relating treatment duration to renal outcomes.


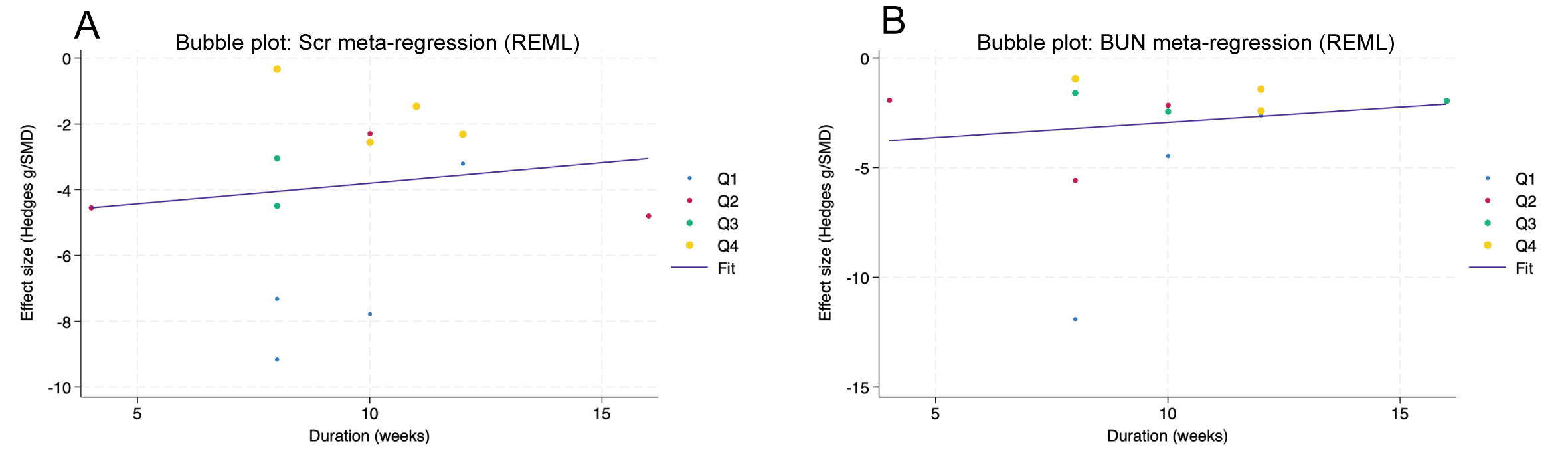


**Note.** Bubble plots show study-level effect sizes (Hedges’ g) versus treatment duration (weeks) for (A) serum creatinine (Scr) and (B) blood urea nitrogen (BUN). The solid line indicates the fitted univariable random-effects meta-regression (REML). Bubble size is proportional to inverse-variance weight (1/SE²) and colors denote weight quartiles (Q1–Q4; lowest to highest). No linear association was observed (Scr: β = 0.13, *P* = 0.64; BUN: β = 0.14, *P* = 0.61).

**Supplementary Table S13**. Univariable random-effects meta-regression (REML) of study-level covariates for serum creatinine (Scr) and blood urea nitrogen (BUN).

| Outcome | *k* | Covariate | β | 95% CI | *P* | Adj R² | I² res |
| --- | --- | --- | --- | --- | --- | --- | --- |
| Scr | 13 | dose (group 2 vs 1) | -0.365 | -3.58 to 2.85 | 0.807 | -11.46% | 84.92% |
|  |  | species (group 2 vs 1) | 2.489 | -0.42 to 5.40 | 0.087 | 19.41% | 84.67% |
|  |  | model type (2 vs 1) | 1.470 | -1.69 to 4.63 | 0.032 | 24.39% | 77.32% |
|  |  | model type (3 vs 1) | 4.638 | -0.42 to 9.69 | 0.068 |  |  |
|  |  | model type (4 vs 1) | 3.506 | -1.56 to 8.57 | 0.152 |  |  |
|  |  | duration | 0.125 | -0.451 to 0.701 | 0.643 | -10.15% | -10.15% |
| BUN | 12 | dose (group 2 vs 1) | -0.105 | -3.59 to 3.38 | 0.948 | -22.04% | 79.73% |
|  |  | species (group 2 vs 1) | 1.288 | -2.53 to 5.11 | 0.470 | -13.60% | 80.41% |
|  |  | model type (2 vs 1) | 1.921 | -1.80 to 5.64 | 0.272 | -10.27% | 81.41% |
|  |  | model type (3 vs 1) | 2.359 | -2.26 to 6.97 | 0.277 |  |  |
|  |  | duration | 0.139 | -0.44 to 0.72 | 0.606 | -20.58% | 80.71% |

**Note.** *k* denotes the number of comparisons. β is the meta-regression coefficient with 95% CI and associated *P* value. Adj R² is the proportion of between-study heterogeneity explained by the covariate; I²res indicates residual heterogeneity after meta-regression. For categorical covariates, group 1 is the reference. Dose: <100 vs ≥100 mg/kg; Duration: <10 vs ≥10 weeks; Species: mice vs rats; DN model: STZ, STZ+HFD, STZ + unilateral nephrectomy, db/db.

**Supplementary Table S14**. Assessment of small-study effects and publication bias.

| Outcome | *k* | Begg's test (*P*) | Egger's test (*P*) | Funnel plot (visual inspection) | Trim-and-fill imputed studies (n) | Trim-and-fill adjusted pooled effect (Hedges’ g) (95% CI) | Interpretation |
| --- | --- | --- | --- | --- | --- | --- | --- |
| BG | 8 | — | — | Asymmetric | — | — | Possible small-study effects |
| Scr | 13 | < 0.001 | < 0.001 | Asymmetric | 0 | -3.88 (-5.21, -2.55) | Possible small-study effects |
| BUN | 12 | < 0.001 | 0.002 | Asymmetric | 0 | -2.98 (-4.28, -1.67) | Possible small-study effects |
| KI | 8 | — | — | Asymmetric | — | — | Possible small-study effects |

**Note.** Begg’s and Egger’s tests were performed only when *k* ≥ 10; otherwise, “—” is shown. When asymmetry was suggested and *k* ≥ 10, trim-and-fill (random-effects model; REML) was applied; therefore, it was not applied to BG or KI. ***Abbreviations:*** BG, blood glucose; Scr, serum creatinine; BUN, blood urea nitrogen; KI, kidney index.
